# Doping Engineering in Manganese Oxides for Aqueous Zinc-Ion Batteries

**DOI:** 10.3390/ma17133327

**Published:** 2024-07-05

**Authors:** Fanjie Ji, Jiamin Yu, Sen Hou, Jinzhao Hu, Shaohui Li

**Affiliations:** School of Materials Science and Engineering, Zhengzhou University, Zhengzhou 450001, China; fanjieji@stu.zzu.edu.cn (F.J.); yjm3594943185@gs.zzu.edu.cn (J.Y.); senhou@stu.zzu.edu.cn (S.H.); hujinzhao2021@stu.zzu.edu.cn (J.H.)

**Keywords:** manganese oxides, doping engineering, cathode materials, aqueous zinc-ion batteries

## Abstract

Manganese oxides (Mn_x_O_y_) are considered a promising cathode material for aqueous zinc-ion batteries (AZIBs) due to their high theoretical specific capacity, various oxidation states and crystal phases, and environmental friendliness. Nevertheless, their practical application is limited by their intrinsic poor conductivity, structural deterioration, and manganese dissolution resulting from Jahn–Teller distortion. To address these problems, doping engineering is thought to be a favorable modification strategy to optimize the structure, chemistry, and composition of the material and boost the electrochemical performance. In this review, the latest progress on doped Mn_x_O_y_-based cathodes for AZIBs has been systematically summarized. The contents of this review are as follows: (1) the classification of Mn_x_O_y_-based cathodes; (2) the energy storage mechanisms of Mn_x_O_y_-based cathodes; (3) the synthesis route and role of doping engineering in Mn_x_O_y_-based cathodes; and (4) the doped Mn_x_O_y_-based cathodes for AZIBs. Finally, the development trends of Mn_x_O_y_-based cathodes and AZIBs are described.

## 1. Introduction

The exhaustive use of traditional energy sources, such as coal and fossil fuels, has not only depleted traditional energy reserves but also caused significant environmental pollution. Therefore, there is an imperative requirement to develop clean and renewable energy resources, including solar energy, wind energy, ocean energy, and biomass energy. However, these new energy sources have time and space discontinuity, which limits their widespread application. Therefore, efficient energy conversion and storage systems are required [1,2,3,4]. Rechargeable batteries are considered as the most promising candidates due to their excellent energy efficiency, long cycle life, cost-effectiveness, and environmental friendliness [5,6,7,8]. So far, rechargeable batteries utilizing various charge carriers, such as Li^+^, Na^+^, K^+^, Ca^2+^, Mg^2+^, Zn^2+^, and Al^3+^, accompanied by either organic or aqueous electrolyte have been reported [9,10,11]. Although a high energy density results from the wide electrochemical window in the organic electrolyte, the toxicity, flammability, and volatility, which pose serious safety hazard and environmental risks, limit the practical application of non-aqueous batteries [12]. In contrast, aqueous batteries using water as the electrolyte offer several advantages, consisting of simple assembly process, extended service life, enhanced safety, environmental friendliness, and affordability. Importantly, the higher ionic conductivity of the aqueous electrolyte than that of the organic electrolyte can grant aqueous batteries a superior rate performance and fast charging characteristics [13,14,15].

Among these ion batteries, AZIBs have several advantages: (i) high ionic conductivity in aqueous electrolyte [16]; (ii) reversible electrodeposition of the zinc anode [17]; (iii) low redox potential (−0.76 V vs. SHE); (iv) high gravimetric capacity (820 mAh g^−1^) and volume specific capacity (5851 mAh cm^−3^) [18]; (v) excellent stability of the zinc anode in neutral solution [17]; (vi) non-volatile and non-toxic aqueous electrolyte [19]; and (vii) abundant zinc resources contributing to a low cost [20]. Nevertheless, the commercial application of AZIBs is hampered by the scarcity of substances capable of reversible Zn^2+^ storage upon extended cycling, as well as the structural collapse of cathodes. As the primary host for the insertion and extraction of Zn^2+^, cathode materials are a crucial factor in influencing the electrochemical performance of AZIBs [21]. To address these challenges, extensive research efforts have been devoted to developing high-electrochemical-performance cathode materials for AZIBs.

Currently, cathode materials in AZIBs can be divided into five categories: manganese-based compounds [22], vanadium-based oxides and vanadates [23], Prussian blue analogues [24], organic compounds [25], and metal chalcogenides [26]. Vanadium-based materials are prone to collapse after long-term cycling. Otherwise, they have a number of problems to be solved, including potential toxicity, slow kinetics for Zn^2+^ insertion, and a low operating voltage. Prussian blue analogues with inherently low specific capacity tend to be easily oxidized under high potentials, resulting in rapid specific capacity degradation during cycling. In general, organic compounds have poor crystallinity or amorphous structure, an unsatisfactory output voltage, and a poor rate performance and cycling stability. Transition metal sulfides are affected by problems such as serious volume expansion, poor conductivity, and low discharge voltage, which hinder their practical application. Among them, manganese-based compounds have diverse valence states and crystal phases, and a series of redox reactions during charging/discharging cycles provide optional capacities and voltage platforms [27,28,29]. In addition, manganese-based compounds possess a stable tunneling structure and a three-dimensional spatial framework, facilitating the sufficient accommodation of Zn^2+^ [30,31,32] and ensuring the acceptable operating voltage and high theoretical capacity of batteries. Thus, in the past few years, manganese-based materials have been widely utilized as cathodes for AZIBs and have become a research hotspot [33,34]. However, manganese-based materials have several drawbacks to overcome, including Mn^3+^ displacement and Mn dissolution induced by the Jahn–Teller effect, structural changes inducing capacity decay and cycle life reduction, and low ionic conductivity inducing poor rate performance and unsatisfactory capacity [35,36,37]. Therefore, it is imperative to propose solutions to improve the electrochemical performance of Mn_x_O_y_ cathodes and promote their practical application in AZIBs.

Given the shortcomings of Mn_x_O_y_ cathodes, various strategies have been exploited to improve their electrochemical performance, including defect engineering, doping engineering, interface engineering, pre-intercalation engineering, and morphology controlling. Compared to other modification strategies, doping engineering can improve the electrochemical performance of AZIBs by boosting electron and ion conductivity, expanding the availability of electrochemical active sites, accelerating the reaction kinetics, and ensuring the longevity of the structural integrity [36,37]. As a common and widely used modification strategy, doping engineering involving cation doping and anion doping has attracted extensive research interest. Doping engineering is utilized to alter the electrical, magnetic, optical, mechanical, and thermal properties of materials by manipulating their charge and spin distribution and band gap [37,38,39]. For Mn_x_O_y_ cathodes, doping engineering can modulate their intrinsic crystal structure, charge and ion state, and band gap and further influence their electrochemical performance [38,39]. In particular, the Zn^2+^ storage performance of Mn_x_O_y_ is closely related to the composition, doping level and position, and bonding configuration of the dopants. Over the past few decades, great progress has been made in doped Mn_x_O_y_-based cathodes, and numerous synthesis routes have been developed. However, most of the currently reported methods are complex and expensive for grid-scale production; a feasible and inexpensive method needs to be further explored before the commercialization of AZIBs.

In recent years, numerous reviews have examined Mn_x_O_y_-based cathodes for AZIBs, including MnO_2_-based cathodes [38,39,40,41,42] and the crystal structure, energy storage mechanisms, and modification strategies of Mn_x_O_y_-based cathodes [43,44,45,46,47,48,49]. However, a comprehensive and systematic summary of the beneficial effects of doping on the electrochemical performance of Mn_x_O_y_-based cathodes for AZIBs is still needed. In addition, a systematic categorization and in-depth analysis concerning the energy storage mechanisms within doping-enhanced Mn_x_O_y_-based cathodes for AZIBs is still lacking. Based on the different oxidation states and crystal structures, this review first focuses on the classification of Mn_x_O_y_-based cathodes. Then, the charge storage mechanisms, the existing problems, and the corresponding optimization strategies for Mn_x_O_y_ cathodes are discussed in detail. In addition, according to different compositions (MnO, MnO_2_, Mn_2_O_3_, and Mn_3_O_4_) and crystal phases (*α*-, *δ*-, *β*-, *ε*-, and *γ*-MnO_2_), the doping technology, electrochemical performance, and inherent improvement mechanisms of doped Mn_x_O_y_ cathodes are comprehensively and incisively elaborated. Finally, valuable research directions for Mn_x_O_y_ cathodes and AZIBs are prospected.

## 2. Classification of Mn_x_O_y_-Based Cathodes

**Figure 1:**
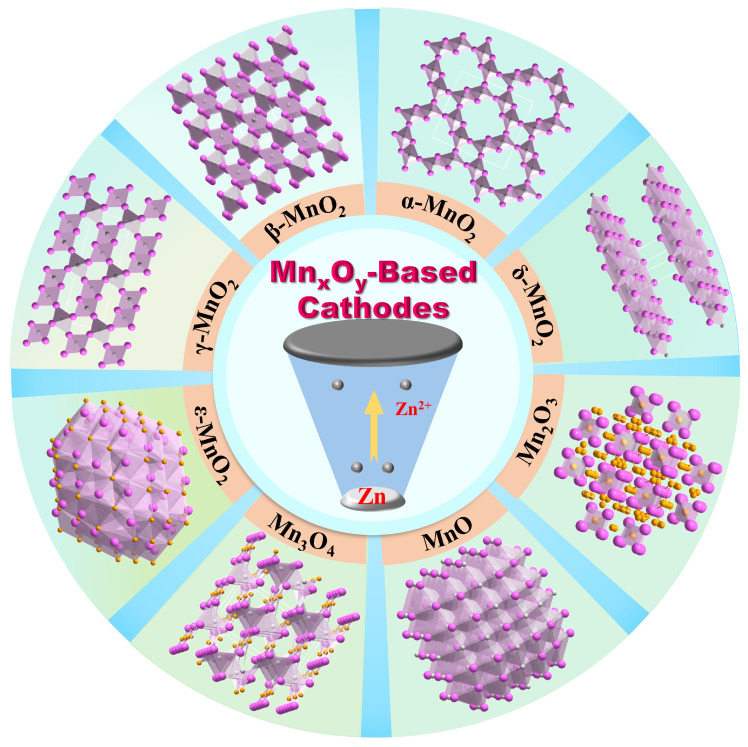
Schematic diagram of diverse crystal structures of Mn-based oxides, containing *α*-, *β*-, *δ*-, *γ*-, and *ε*-MnO_2_, MnO, Mn_2_O_3_, and Mn_3_O_4_.

As shown in Figure 1, there are many forms of Mn_x_O_y_, including MnO_2_, MnO, Mn_2_O_3_, and Mn_3_O_4_, in which MnO_2_ consists of several crystal structures, containing *α*-, *β*-, *γ*-, *δ*-, *ε*-, *λ*-, *T*-(perovskite MnO_2_), and *R*-MnO_2_. The octahedral MnO_6_ unit is the basic building block for all MnO_2_ crystal forms. Due to the advantages of various Mn_x_O_y_ structures, it has been widely investigated as a promising cathode in AZIBs in recent years. As an optimization strategy, doping engineering can tailor the electrical, magnetic, optical, mechanical, and thermal properties of Mn_x_O_y_ by manipulating their charge and spin distribution and band gap, thus boosting the electrochemical performance. Previous works have confirmed that the electrochemical performance of Mn_x_O_y_ (e.g., MnO, MnO_2_, Mn_2_O_3_, and Mn_3_O_4_) can be improved after cation or/and anion doping due to their effect on the average valence, crystalline phase, and structure [50,51,52,53,54]. In this section, different phases and structures of Mn_x_O_y_ are summarized, which can help to understand the mechanism after ion doping.

### 2.1. α-MnO_2_

*α*-MnO_2_ has a one-dimensional [2 × 2, ~0.46 nm × 0.46 nm] tunnel structure, which belongs to the body-centered tetragonal crystal system and the *I4*/*m* space group. The large pore size of *α*-MnO_2_ enhances the diffusion performance of Zn^2+^ ions within the matrix framework. This allows for effective storage and the rapid transfer of guest cations following the z-axis direction [37,40,49].

### 2.2. β-MnO_2_

*β*-MnO_2_ has a [1 × 1, ~0.23 nm × 0.23 nm] tunnel structure with a tunnel size of 2.3 × 2 Å. Its numerous opening channels are capable of holding massive cations [43]. However, *β*-MnO_2_ also has its limitations: the narrowness of its tunnel structure restricts it to providing only a modest capacity [40].

### 2.3. γ-MnO_2_

*γ*-MnO_2_ is a rhombohedral crystal system composed of alternating [1 × 1, ~0.23 nm × 0.23 nm] channels of pyrolusite and [1 × 2, ~0.23 nm ×0.46 nm] channels of rhodochrosite. It comprises MnO_6_ octahedral units which are interconnected through sharing edges and corners. Each unit cell contains four MnO_2_ molecules [38]. Furthermore, *γ*-MnO_2_ is a hybrid structure that contains both single- and double-chain configurations, and it is often regarded as an intermediate form between *β*-MnO_2_ and *R*-MnO_2_ [40].

### 2.4. δ-MnO_2_

*δ*-MnO_2_ is composed of corner-sharing MnO_6_ octahedra and corresponds to the monoclinic crystal phase with the *P2*/*m* space group. Additionally, it exhibits a representative two-dimensional laminar structure characterized by a substantial inter-lamellar spacing of approximately 7 Å; this feature allows for an increased number of active sites that facilitate the intercalation and de-intercalation of Zn^2+^. The layered structure of *δ*-MnO_2_ is constructed from MnO_6_ octahedral sheets that grow along shared edges. To enhance the stability of this layered architecture, interlayer spaces are occupied by water molecules and cations [40].

### 2.5. ε-MnO_2_

*ε*-MnO_2_, similar to *γ*-MnO_2_, is also referred to as hexagonal pyrolusite. Its manganese lattice is highly disordered, and the tunnels within its structure are irregularly shaped. In addition, *ε*-MnO_2_ has a metastable phase containing edge-shared MnO_6_ octahedra, where Y represents vacancies. This arrangement hinders the rapid intercalation and de-intercalation of ions and protons, which is essential for efficient energy storage. The inherent low electrochemical activity, coupled with poor conductivity, yields suboptimal electrochemical characteristics.

### 2.6. MnO

MnO consists of a MnO_6_ octahedron and lacks a tunnel structure, making it electrochemically inactive. However, the addition of Mn defects is able to create pathways for the insertion of Zn^2+^, thereby enhancing the conductivity of MnO [40].

### 2.7. Mn_2_O_3_

The crystal structure of Mn_2_O_3_ is devoid of both tunnel structures and expansive interlayer spacing. Mn^3+^ is situated in the octahedral coordination, with four Mn ions encircling each oxygen ion. Furthermore, a reversible phase transition reaction takes place between Mn_2_O_3_ and birnessite during the cycling.

### 2.8. Mn_3_O_4_

Mn_3_O_4_ is a multivalent manganese oxide featuring a spinel structure, with the chemical formula Mn^II^Mn^III^_2_O_4_. It incorporates both Mn^2+^ and Mn^3+^ valence states. Mn^2+^ is located in the tetrahedral (4a) sites, while the Mn^3+^ occupies the octahedral (8d) sites within an intermediate, slightly twisted cubic close-stacked array of oxygen atoms [43]. Moreover, Mn_3_O_4_ is also recognized as an outstanding cathode material due to its excellent theoretical capacity.

## 3. Energy Storage Mechanisms of Mn_x_O_y_-Based AZIBs

**Figure 2:**
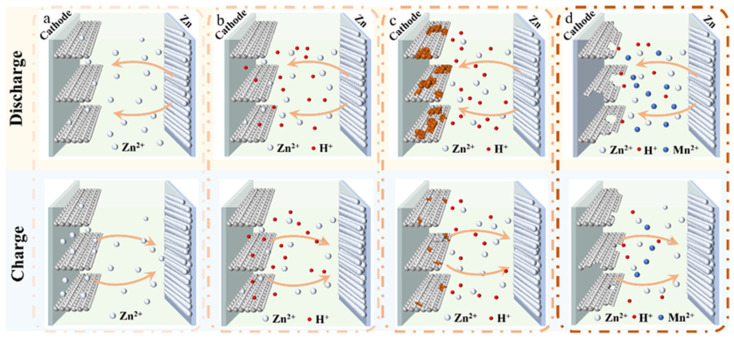
Schematics diagram of energy storage mechanism classification of AZIBs based on different reaction mechanisms. (**a**) Reversible Zn^2+^ insertion mechanism. (**b**) Zn^2+^/H^+^ co-insertion mechanism. (**c**) Conversion reaction mechanism. (**d**) Dissolution/deposition mechanism.

The in-depth study of the energy storage mechanisms can effectively guide the optimization of materials’ performance, making it a core focus in the field of energy storage materials. However, due to the diverse crystal structure of Mn_x_O_y_ and the influence of the electrolyte, the current charge storage mechanisms of AZIBs are full of controversies, and there is no generally accepted theory. Based on the latest reported works, there are four types of reaction mechanisms (Figure 2), including Zn^2+^ intercalation/de-intercalation, reversible Zn^2+^ and H^+^ co-intercalation/de-intercalation, the chemical conversion reaction, and dissolution/deposition.

### 3.1. Zn^2+^ Insertion Mechanism

The Zn^2+^ insertion mechanism belongs to the earliest and most important mechanism in AZIBs. Like the insertion mechanism existing in traditional alkali-metal-ion batteries, Zn^2+^ can be easily inserted/extracted from manganese-based materials during the charging/discharging process (Figure 2a). However, due to the different crystal phases of manganese-based materials, the Zn^2+^ insertion mechanism often manifests in a more complex reaction pathway [55]. For example, in the case of Mn_x_O_y_ with different crystal phases, although they will gradually evolve into a Mn_x_O_y_-layered phase with interlayer water molecules on account of structural transformation during the redox reaction, the evolution process is partially different [56]. For example, in the case of *α*-MnO_2_ with 1 × 1 or 2 × 2 tunnel structures, Kang’s group [57] observed a structural evolution process where Mn^4+^ is reduced to Mn^3+^. Then, the Mn^3+^ is dissolved into the electrolyte through chemical disproportionation, and finally, the Zn–Bussel mine is obtained. For *γ*-MnO_2_ with alternating pyrolusite and rhodochrosite channels, various intermediates including ZnMn_2_O_4_, tunnel *γ*-Zn_x_MnO_2_, and layered Zn_y_MnO_2_ may appear successively during its evolution [58].

### 3.2. Zn^2+^/H^+^ Co-Insertion Mechanism

The co-intercalation/de-intercalation mechanism of Zn^2+^ and H^+^ involves the transport of two charge carriers into the skeleton of the manganese-based material, which differs significantly from the Zn^2+^ insertion mechanism (Figure 2b). Due to the smaller size and weaker electrostatic interaction of H^+^ compared to Zn^2+^, the insertion thermodynamics and kinetics of the two charge carriers are completely different [33,59,60]. It is generally believed that the interaction between Zn^2+^ and MnO_2_ occurs through the intercalation process, while the interaction between H^+^ and MnO_2_ occurs through the chemical conversion reaction [37,61,62,63]. During the discharge process, H^+^ and Zn^2+^ are incorporated into the manganese-based material to form MnOOH and Zn_x_MnO_2_, respectively, and then released during the subsequent charging process, which together form a reversible electrochemical process. However, due to the different reaction kinetics, researchers have different opinions on the order of H^+^ and Zn^2+^ intercalation reactions [37,64,65]. Many scholars have conducted related research using different MnO_2_ materials and provided various evidence regarding the controversy over the insertion sequence, but no unified conclusion has been reached yet.

### 3.3. Conversion Reaction Mechanism

The chemical conversion reaction mechanism is distinct from the insertion mechanism of Zn^2+^, in which the insertion/extraction of Zn^2+^ cannot contribute to the battery capacity (Figure 2c) [66,67,68,69]. Oh et al. [70] proposed that during the discharge process, the Mn element in MnO_2_ is first electrochemically reduced to Mn^3+^ and then dissolved in the electrolyte as Mn^2+^ through a disproportionation reaction. Therefore, the generation of Zn_4_SO_4_(OH)_6_·5H_2_O and the reduction–disproportionation–dissolution of Mn mainly contribute to its capacity. However, more scholars believe that the chemical conversion mechanism refers to the reversible electrochemical reaction between MnO_2_ and MnOOH/Zn_4_SO_4_(OH)_6_·5H_2_O [63,67,71]. In the discharge process, MnO_2_ is transformed into MnOOH by interacting with the protons in the solution, and the OH^−^ generated by the water ionization combines with Zn^2+^ and SO_4_^2+^ in the electrolyte to precipitate Zn_4_SO_4_(OH)_6_·5H_2_O [70,72]. Given the scarcity and controversy of reports on the chemical conversion mechanism, most work focuses on the embedding mechanism of Zn^2+^ as the electrochemical reaction mechanism.

### 3.4. Dissolution/Deposition Mechanism

The dissolution/deposition mechanism exhibits a significant correlation with the composition of the electrolyte. In short, layered MnO_2_ and Mn^2+^ undergo a reversible dissolution/deposition process during charging/discharging (Figure 2d). Different from the mechanism that is mainly based on a single-electron redox reaction, the dissolution/deposition mechanism represents a new redox chemistry that relies on a two-electron transfer reaction, which is a crucial factor for improving the battery capacity [27,73,74]. Kundu et al. [75] carried out extensive research on the electrolyte of *α*-MnO_2_ and found that when the electrolyte does not contain additives that can form a layered hydroxide (Zn_4_SO_4_(OH)_6_·5H_2_O), the capacity of the battery will be significantly attenuated. Similarly, Jaekook Kim et al. [76] found that when the electrolyte contains both ZnSO_4_ and MnSO_4_ as additives, it is beneficial for the reversible electrodeposition/dissolution of Mn^2+^ on the surface of the cathode and formation of the surface MnO_2_ layer or bulk phase formed by reversible Zn^2+^ insertion, leading to excellent structural stability and high reversibility. Liang et al. [77] conducted a capacity test in single ZnSO_4_ electrolyte and found that its discharge capacity was significantly attenuated, speculating that the dissolution/deposition mechanism controls the energy storage process.

## 4. Doping Engineering of Mn_x_O_y_-Based Cathodes

### 4.1. Synthesis Route of Heteroatom-Doped Mn_x_O_y_-Based Cathodes

An essential step in optimizing the electrochemical performance of doped manganese oxides is the synthesis process of doped manganese oxides. The methods for synthesizing doped manganese oxides are diverse, and the following section presents several widely used approaches.

#### 4.1.1. Hydrothermal Method

**Figure 3:**
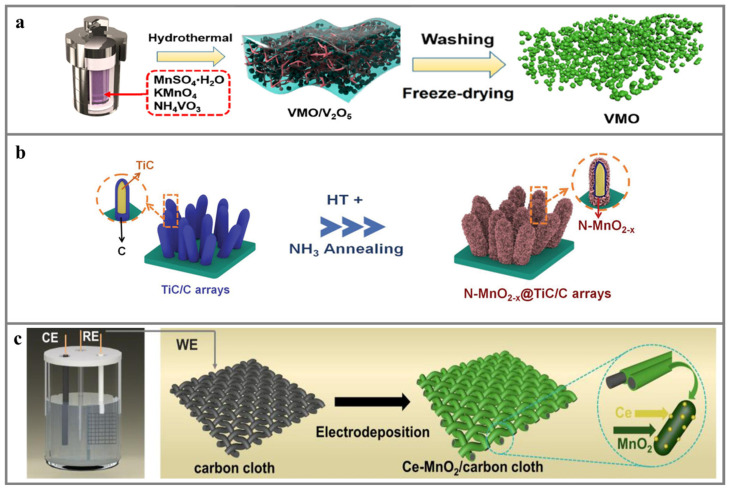
(**a**) Schematic of hydrothermal method to prepare V-doped MnO_2_ [80]. Copyright © 2021, American Chemical Society. (**b**) Schematic of heat treatment method to prepare N-doped MnO_2−x_ [81]. Copyright © 2019 Wiley. (**c**) Schematic of electrodeposition method to prepare Ce-doped MnO_2_ [82]. Copyright 2022, Elsevier.

The hydrothermal method is the most commonly used method due to its ease of control, allowing for the production of nanoparticles with tailored morphologies, easy control of ion doping, and the synthesis of a wide range of phase structures. In a study by Li et al. [78], stannous chloride (SnCl_2_) was utilized as the dopant, with potassium permanganate (KMnO_4_) and manganese sulfate monohydrate (Mn(SO_4_)·H_2_O) serving as the manganese sources. The pH of the solution was meticulously adjusted with hydrochloric acid. Subsequent hydrothermal processing at 180 °C for 24 h yielded a uniform rod-like structure of *α*-MnO_2_. Yan et al. [79] employed aluminum nitrate (Al(NO_3_)_3_) as a heteroatom dopant. The black aluminum was pre-embedded into the MnO_2_ matrix through a one-step hydrothermal process, which involved a continuous reaction at 140 °C for 4 h. The resulting material not only exhibits superior zinc storage performance but also displays a distinctive morphological structure. It is characterized by a 3D sea-urchin-like hollow microsphere with a size of 4.5 to 5.0 µm. Li et al. [80] reported an innovative strategy for the preparation of V-doped MnO_2_ using a hydrothermal method with an excess of dopant. As depicted in Figure 3a, a VMO/V_2_O_5_ hydrogel monolithic column containing V_2_O_5_ precipitates was produced by reacting a mixture of ammonium metavanadate (NH_4_VO_3_) and manganese salt in an autoclave for 12 h. After thorough washing with copious amounts of deionized water and the subsequent dissolution of the V_2_O_5_, the final product was V-doped MnO_2_. Though the hydrothermal method has many advantages, commercialization is still limited by the high energy demands, high equipment requirements, and long reaction times, which increase the product costs.

#### 4.1.2. Co-Precipitation Method

Co-precipitation is widely used due to its significant advantages: the resulting products exhibit uniform mixing and a short synthesis time and their morphology, particle size, and properties can be finely tuned by changing the precipitation parameters. Lu and colleagues [81] employed the co-precipitation method to prepare a La-Ca co-doped *ε*-MnO_2_ cathode (LCMO). The urchin-like nanostructure provides numerous active sites for Zn^2+^. The dual-ion doping strategy enlarges the tunnel diameter of MnO_2_, effectively lowering the energy barrier for Zn^2+^ diffusion. In addition, the LCMO exhibits enhanced conductivity and a more stable crystalline structure, which significantly boosts its electrochemical performance. Dong and team [82] synthesized an aerogel-structured MnO_2_ composed of defective ultrathin nanosheets using a simple co-precipitation method augmented with V_2_O_5_ gel. Vanadium doping facilitated the creation of abundant oxygen vacancies and the assembly of an aerogel morphology from ultrathin nanosheets. The presence of V doping and oxygen vacancies can modulate the electronic structure, thereby enhancing the conductivity and lowering the Zn^2+^ diffusion energy barrier, which in turn improves the electrochemical performance. Although the co-precipitation method offers considerable advantages and has some industrial applications, it still had some drawbacks that needed to be overcome, including the presence of pH gradients resulting from the ineffective stirring, the broad particle size distribution of the products, and extra complex high-temperature treatment to eliminate the impurities.

#### 4.1.3. Ball Milling

The synthesis of doped manganese oxide through ball milling involves the thorough mixing of manganese oxide with the dopant, followed by the introduction of the mixture into a ball mill. The addition of an appropriate amount of grinding media facilitates the milling process, ultimately yielding the desired doped manganese oxide. This method is favored for its simplicity, ease of operation, cost effectiveness, and the uniformity of raw material mixing. For example, Sun et al. [83] discovered that simple wet ball milling could incorporate nitrogen atoms into MnO_2_ for the first time. Using MnO_2_ powder, urea particles, and deionized water as precursors, samples with and without urea particles were prepared to investigate the effect of nitrogen doping. It was concluded that interstitial nitrogen-doped MnO_2_, in conjunction with oxygen vacancies, exhibits an increased adsorption capacity for H^+^, which in turn affects the electrochemical performance of the cathode. However, the milling easily introduces impurities during the milling process, and excessive milling can lead to lattice distortion or amorphization, which adversely affects the performance of the material.

#### 4.1.4. Calcination Treatment

The calcination treatment is simple and easy to scale up, which can provide high kinetics for guest ion intercalation and make it accessible for various applications. Xia et al. [84] reported an N-doped MnO_2−x_ dendritic structure cathode by calcining MnO_2_ in an NH_3_ atmosphere at a low temperature of 200 °C. As depicted in Figure 3c, MnO_2_ nanosheets were initially deposited on a TiC/C framework using a hydrothermal approach. The resulting MnO_2_@TiC/C was then annealed in an NH_3_ atmosphere. Similarly, Sun et al. [35] prepared an S-doped MnO_2_ by calcining MnO_2_ under S vapor at the temperature of 450 °C. The S doping can moderate the electronic conductivity, reduce the electronegativity of MnO_2_, and debilitate the electrostatic interactions with Zn^2+^, thus boosting the electrochemical performance. However, due to the surface reaction, the doping usually occurs near the surface of MnO_2_, which can hardly be homogeneously doped at the atomic level, and the doping content cannot be precisely controlled. Thus, the calcination treatment is usually combined with the hydrothermal method, co-precipitation method, and sol–gel method as a post-treatment. In other words, precursors are first synthesized and then calcined to form final products.

#### 4.1.5. Sol–Gel Process

Different from the direct calcination method, the sol–gel method is a wet chemical technique in which a precursor containing doped ions can be mixed homogeneously at the atomic level. Thus, the sol–gel technique is lauded for its distinctive ability to regulate the morphology and structure of the material by fine-tuning the synthesis parameters, yielding products of high purity, nanosize, and well-controlled stoichiometry. Xiang’s team [85] introduced an enhanced sol–gel approach for the synthesis of Na_0.44_MnO_2_/Mn_2_O_3_ composites. They initiated the process by mixing a manganese acetate solution with a sodium citrate solution to form metal chelates. To facilitate the coordination of manganese ions and limit their hydrolysis rate, the pH of the mixture was delicately adjusted with an ammonium hydroxide solution. After a reaction period of 4 h at 80 °C, a loose precursor was obtained through freeze-drying, which was then subjected to a calcination process to yield the final composite material. Parkin et al. [86] prepared potassium permanganate and D (+)-glucose solutions separately. After rapid mixing and subsequent cooling, a brown gel was formed. This gel, once dried and calcined at 400 °C, was transformed into sodium birnessite (K_0.28_MnO_2_). Unfortunately, the sol–gel method usually needs to use organic acids as chelating agents, and the decomposition of the organic component, solvent evaporation, and gelation require extra energy consumption, which will increase the cost of the product.

#### 4.1.6. Other Methods

Apart from the above-summarized synthesis methods, several other strategies, including the ion exchange method and electrodeposition method, have also been reported in recent years to construct heteroatom-doped Mn_x_O_y_ materials. For example, Dai et al. [87] synthesized a porous H_x_Mn_2_O_4_ cathode material by using a cation exchange method. They first prepared ZnMn_2_O_4_ by coupling the co-precipitation and calcination treatment methods. After soaking ZnMn_2_O_4_ in 0.5 M H_2_SO_4_, the Zn^2+^ ions in a ZnMn_2_O_4_ template were substituted by H^+^ through the Jahn–Teller disproportionation reaction of the Mn^3+^ ions and ZnO_4_ tetragonal distortion, yielding a distinctive crystal structure with remarkable electrochemical performance. Liu et al. [88] prepared a Ce-doped MnO_2_ cathode by utilizing acetylene-black-modified carbon cloth as the substrate for the electrodeposition. After the electrodeposition, the Mn^2+^ and Ce^3+^ ions in the electrolyte were oxidized and coated on the surface of carbon cloth fibers. The presence of Ce ions can enlarge the Zn^2+^ transport channel, accelerate the ion/electron migration, and stabilize the structure, thus improving the electrochemical performance.

### 4.2. The Role of Doping Engineering

Currently, in order to promote the commercialization of Mn-based cathodes, various modification technologies have been proposed to address their drawbacks, including defect engineering [47,89,90], pre-embedding engineering [91,92,93,94], surface engineering [95,96,97], and composite construction [98,99,100]. The fabrication of defects can significantly enhance the capacity and reaction kinetics of cathodes by providing additional sites and regulating the electron and crystal structure. However, the prevailing research mainly focuses on single defects, in which it is difficult to achieve precise control of the defect site and concentration. In addition, it has not been determined whether the introduction of defects will adversely affect the crystal structure during battery cycling. Pre-embedding engineering refers to the insertion of other atoms or small molecules into electrode materials, which can enlarge the interlayer spacing, reinforce the crystal phase, and enhance conductivity. However, the most commonly used method for pre-embedding is hydrothermal technology, which is incompatible with large-scale manufacturing and rapid preparation. In addition, pre-embedded engineering is not universal, and a large number of pre-embedded intercalation agents could alter the layer structure and realign the main layer, resulting in the generation of non-layered fresh phases and the loss of the inherent advantages of the layer structure. Surface engineering refers to coating the electrode material with other highly conductive materials, including graphene, carbon nanotubes, MXenes, conductive polymers, and metal oxides, to improve the conductivity and increase the active sites in the cathode, as well as to protect the material. It is a popular strategy to solve problems including the poor reaction kinetics, structural collapse, and cathode dissolution of Mn. However, the transfer kinetic relationship between the electrolyte and the coating layer, the reaction mechanism, and the changes in physical and chemical properties need to be further investigated. Different from surface engineering, the composite construction strategy offers more patterns, including laminated, core–shell, and sandwich structures. Although the combination of a manganese-based cathode and coating layer is capable of imparting new properties to the cathode and enhancing the advantages of single materials, it also presents challenges such as reducing the volume energy density and increasing the cost.

**Figure 4:**
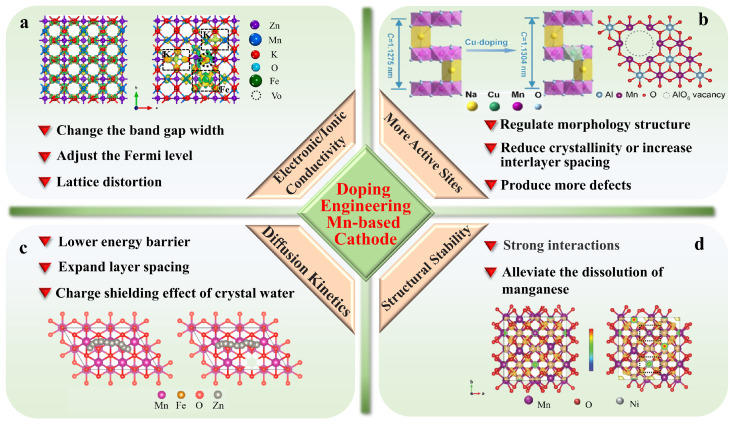
Overview of doping engineering for performance improvement in manganese-based metal oxides. (**a**) Enhancing intrinsic electron/ion conductivity. (**b**) Increasing electrochemical active sites. (**c**) Promoting diffusion kinetics. (**d**) Maintaining structural stability.

Doping engineering can adjust the lattice parameters, strengthen the lattice structure, and alleviate the structural damage and untoward reactions through inhibiting cation mixing, lattice distortion, ion migration, etc. [101,102,103,104]. Specifically, heteroatom doping can not only induce unit cell expansion, improving the ion transport path, but can also induce charge redistribution, promoting electron migration. Based on the structure–activity relationship between the doping strategy and the performance of AZIBs, four aspects are classified (Figure 4): enhancing the electron and ion conductivity, increasing the electrochemical active sites, accelerating the reaction kinetics, and maintaining the stability of the structure [105,106,107].

#### 4.2.1. Enhancing Intrinsic Electron/Ion Conductivity

The introduction of other elements into the manganese-based cathode may change its charge and spin distribution, as well as the band gap, resulting in a significant change in its intrinsic conductivity (Figure 4a) [108]. Zhou et al. [109] used first-principles calculations to investigate the change in the density of states of *α*-MnO_2_ with a 1 × 1 × 3 periodic supercell and found that V doping increases the Fermi level of *α*-MnO_2_ and shifts it toward the bottom of the conduction band. In addition, the band gap is narrowed due to the generation of impurity peaks. Therefore, it is found that the introduction of V increases the conductivity of MnO_2_. Rao et al. [110] investigated the diffuse reflectance spectra of Zn-doped *δ*-MnO_2_ and found that with an increasing Zn content, the band gap increases from 2.3 eV (5 mol % doping) to 2.37 eV (10 mol % doping). This variation in the band gap has a strong correlation with the change in the Zn doping amount, which ultimately manifests in an increase in the overall electronic conductivity. Song et al. [111] discovered that the incorporation of Co into MnO_2_ can introduce holes and improve the conductivity by introducing a new electronic state around the Fermi level, which facilitates the electron migration between Mn^4+^ and Mn^3+^, enhances the concentration of redox active sites, and ultimately improves the contribution rate of pseudocapacitance in MnO_2_.

#### 4.2.2. Increasing Electrochemical Active Sites

Increasing the number of electrochemically active binding sites by doping treatment is mainly achieved from two aspects (Figure 4b). First, the introduction of heteroelements can activate more active sites for redox reactions and plug in pseudocapacitance [106,112]. Sun et al. [35] found that the doping of S in MnO_2_ under non-equilibrium conditions, taking high temperature and surface sites as examples, can generate a large number of oxygen defects within the structure or further create an amorphous phase on the surface or edge. On the one hand, this can improve the ion and electron transfer within the structure of the cathode. On the other hand, the amorphous region accelerates the ion transfer in the electrolyte/electrode interface, provides numerous pseudocapacitive active sites, and contributes to the capacity. Zhao et al. [113] found that the Zn doping can change the crystal structure of MnO_2_, referring to more atomic dislocations observed by transmission electron microscopy, which are used as active sites for ion absorption in order to accelerate the electrochemical activity of the active material. Moreover, in the process of preparing manganese-based cathodes, the introduction of impurity elements can induce a change in the morphology and structure, which increases their specific surface and provides abundant active sites for electrochemical reactions.

#### 4.2.3. Promoting Diffusion Kinetics

The electrostatic shielding effect of crystal water can lower the energy barrier, expand the tunnel space of the material, and shorten the diffusion path of electrons and ions; this is favorable in accelerating the diffusion kinetics in manganese-based cathodes (Figure 4c). Fan et al. [114] prepared Na-doped MnO_2_ material by the co-precipitation method and found that the charge shielding effect generated through structural water can promote the rapid transfer of ions within the MnO_2_ crystal. The amorphous characteristics of the cathode also provide more active sites and reduce the ion diffusion pathway. Fang et al. [115] found that the spacing expansion of the tunnel structure in *α*-MnO_2_ originating from K^+^ intercalation consciously generates extra space for the effective transport of H^+^ and Zn^2+^ in the charge/discharge process, ensuring fast diffusion kinetics of the cation. Yan et al. [116] studied the diffusion barrier path with the lowest energy of Zn^2+^ in Fe-doped MnO_2_ and MnO_2_ through density functional theory (DFT) calculations. Apparently, the diffusion of Zn^2+^ along the MnO_2_ cathode encounters the barrier of 380 meV, while the Fe-doped MnO_2_ cathode encounters a lower barrier of 260 meV, indicating that the low-energy-barrier cathode exhibits faster diffusion kinetics than the high-energy-barrier cathode. Compared with MnO_2_, the Fe replacement rearranges the allocation of the electrostatic potential surface, resulting in a topical maximum under an inferior potential, which promotes ion and electron transport, as well as reduces the energy barrier.

#### 4.2.4. Maintaining Structural Stability

Cycle life is one of the most important criteria for evaluating secondary batteries. However, AZIBs have faced the challenge of rapid capacity degradation. The structural damage in the charging/discharging process is thought to be the main reason for the poor cycle life of manganese-based AZIBs, including the dissolution of manganese and irreversible phase transition. Xu et al. [117] obtained uniformly Cu-doped MnO_2_ by the heat treatment of MOF precursors and found that the ratio of Mn^4+^/Mn^3+^ in the undoped sample was lower, indicating that Cu-doped MnO_2_ contains less Mn^3+^ and reduces Mn dissolution and Jahn–Teller distortion. Huang et al. [28] successfully prepared Ni-doped Mn_2_O_3_ by co-precipitation and calcination processes. DFT calculations revealed that the formation energy of Mn_2_O_3_ could be reduced and the Mn-O bond of Mn_2_O_3_ could be effectively stabilized by Ni doping, thus enhancing the structural stability of Mn_2_O_3_. Similarly, Hui et al. [118] found that the doping with low-valent Zn^2+^ replaces the position of Mn and adjusts the electronic structure near the Mn-O bond, thereby accelerating the asymmetric coupling between the O^2−^ and Mn^4+^ and strengthening the structural stability. Therefore, alleviating the dissolution of manganese ions or forming a strong interaction by forming strong ionic bonds is considered to be an efficient strategy to increase the stability of manganese-based oxide cathodes (Figure 4d).

## 5. Doped Mn_x_O_y_-Based Cathodes for AZIBs

### 5.1. MnO_2_

Among the Mn_x_O_y_ materials, MnO_2_ is the most extensively studied cathode in AZIBs [37,119]. MnO_2_ has different crystal structures, mainly consisting of *α*-, *β*-, *γ*-, *δ*- and *ε*-MnO_2_ [120,121,122]. MnO_2_-based cathodes offer several advantages consisting of the natural high abundance of Mn-contributed low cost, low-toxicity-contributed environmental safety, good electrochemical capacity [123,124,125], and capability for multi-electron transfer reactions. However, the commercial application of MnO_2_ cathodes of AZIBs faces challenges such as manganese dissolution, irreversible phase transition, and inferior electronic and ionic conductivity [126]. As one of the effective cathode modification strategies, doping engineering has significant value in enhancing the rate property and cycling stability in MnO_2_ cathodes. Elemental doping serves to alleviate the phase transformation and volume fluctuation in cathodes, thus ensuring their structural stability throughout the cycle. Moreover, doping can introduce defects into the structure, resulting in increased active storage sites for ions and protons. In addition, the lattice expansion and charge redistribution generated by doping are conducive to accelerating the transfer rate of ions and electrons and decrease the electrostatic repulsion between Zn^2+^ and MnO_2_ [35]. According to the difference in crystal types, the synthesis method, electrochemical performance, and internal improvement mechanism of doped *α*-, *δ*-, *β*-, *ε*-, and *γ*-MnO_2_ are summarized and analyzed.

#### 5.1.1. *α*-MnO_2_

The large spacing and stable tunnel structure of *α*-MnO_2_ facilitate the rapid and reversible intercalation/de-intercalation of H^+^ and Zn^2+^, endowing AZIBs with high specific capacity and moderate discharge voltage [127]. However, the slow ion diffusion caused by the poor conductivity of *α*-MnO_2_ and the severe capacity decay and poor rate performance originating from the inevitable dissolution of Mn limit the application of *α*-MnO_2_ in AZIBs. Numerous studies have shown that the ion diffusion kinetics, electronic/ionic conductivity, and structural stability of MnO_2_ can be enhanced through using doping engineering to improve the cycling stability and rate property of the material.

**Figure 5:**
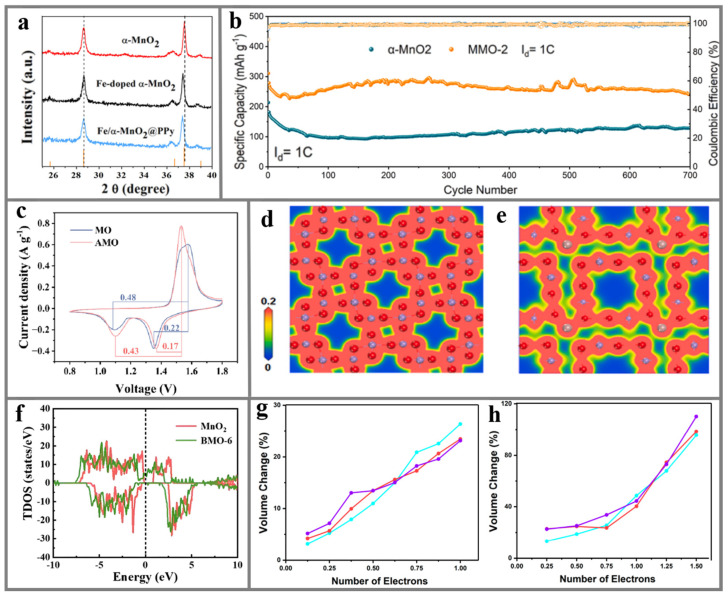
(**a**) The magnified XRD spectra of *α*-MnO_2_, Fe-doped *α*-MnO_2_, and Fe/*α*-MnO_2_@PPy [130]. Copyright 2021, Elsevier. (**b**) The cycle property of bare MnO_2_ and Mg-doped *α*-MnO_2_ (MMO-2) at 1 C [129]. Copyright 2023, Elsevier. (**c**) Comparison of CV curves of Al-doped *α*-MnO_2_ (AMO) and *α*-MnO_2_ (MO) at 0.1 mV s^−1^ [130]. Copyright © 2023, American Chemical Society. (**d**) The distribution maps of charge density in α-MnO_2_ [131]. (**e**) The distribution maps of charge density in Ga-doped *α*-MnO_2_ [131]. Copyright 2023, Elsevier. (**f**) Patterns of total density of states in pristine *α*-MnO_2_ and Bi-doped *α*-MnO_2_ (BMO-6) [132]. Copyright 2022, Elsevier. (**g**) Volume changes with different intercalation steps in MnO_2_H_x_·0.25(H_2_O) (purple line), Mn_0.875_V_0.125_O_2_H_x_·0.25(H_2_O) (red line), and Mn_0.875_Cr_0.125_O_2_H_x_·0.25(H_2_O) (cyan line). (**h**) Volume changes with different intercalation steps in Zn_x_MnO_2_·y(H_2_O) (purple line), Zn_x_Mn_0.875_V_0.125_O_2_·y(H_2_O) (red line), and Zn_x_Mn_0.875_Cr_0.125_O2·y(H_2_O) (cyan line) [133]. Copyright © 2023, American Chemical Society.

In terms of optimizing the reaction diffusion kinetics, heteroatom doping can increase the tunnel size of *α*-MnO_2_ and provide a larger diffusion space for the insertion or de-insertion of Zn^2+^. Xu et al. [128] synthesized polypyrrole-encapsulated and Fe^3+^-doped *α*-MnO_2_ composites by chemical precipitation and acid-catalyzed pyrrole polymerization processes. By comparing the displacement of XRD characteristic peaks, it can be found that the pre-intercalation of Fe^3+^ can increase the interlayer space of *α*-MnO_2_, thereby increasing the rate of Zn^2+^ intercalation/de-intercalation (Figure 5a). Lin et al. [50] introduced Co^2+^ and abundant oxygen vacancies into two-dimensional layered *α*-MnO_2_ nanofibers (Co-*α*-MnO_2_) through the hydrothermal process and plasma technology, respectively. It was found that the doping of Co^2+^ can not only flexibly control the interlayer spacing of MnO_2_ and create more space for the transport of Zn^2+^ but can also enhance the stability of the *α*-MnO_2_-layered skeleton by buffering the volume variation in the charging/discharging process. Therefore, Co-doped *α*-MnO_2_ exhibits excellent rate performance. As current density is restored from 5 to 1 A g^−1^, the cathode still maintains a capacity of 249 mAh g^−1^. The ion diffusion energy barrier can be effectively adjusted by doping engineering to reinforce the ionic diffusion coefficient. Li et al. [129] innovatively synthesized Mg^2+^-doped *α*-MnO_2_ composites with abundant oxygen defects via a simple hydrothermal method. Electrochemical performance characterizations and DFT calculations confirm that the insertion of Mg^2+^ efficiently reduces the charge transfer resistance, polarization, and Zn^2+^ diffusion barrier and improves the structural stability of *α*-MnO_2_. Therefore, the Mg-*α*-MnO_2_ cathode can maintain a high capacity of 311 mAh g^−1^ at 600 mA g^−1^ after 700 cycles (Figure 5b). Additionally, Guo et al. [130] also found that the doping of metallic elements enhances the reaction kinetics of electrode materials by studying the cyclic voltammetry curve of Al-doped MnO_2_. The results demonstrate that Al-doped *α*-MnO_2_ exhibits a more intense oxidation/reduction current than *α*-MnO_2_. Simultaneously, the voltage gap of Al-doped *α*-MnO_2_ (0.17 and 0.43 V) is decreased compared to *α*-MnO_2_ (0.22 and 0.48 V). As displayed in Figure 5c, Al-doped MnO_2_ has more excellent electrochemical reactivity and reaction kinetics.

It is well known that doping engineering can introduce new energy levels and energy band structures or affect the formation of electron–hole pairs, thereby changing the electronic structure in the material and ultimately achieving changes in the ion/electron conductivity of the cathode. Cao et al. [131] constructed Ga-doped *α*-MnO_2_ nanowires and applied them to AZIBs, showing significantly improved electrochemical performance. After 200 cycles under 0.2 A g^−1^, an excellent capacity of 205 mAh g^−1^ can still be obtained. Ga can effectively adjust the electronic distribution of *α*-MnO_2_ and reduce its gap. On the basis of the electron density distribution, it can be seen that Ga doping causes the electron rearrangement of *α*-MnO_2_, resulting in the polarization of the electron cloud of O, thereby improving the conductivity of the material (Figure 5d,e). Similarly, Yuan et al. [132] demonstrated the effect of Bi doping on the conductivity by calculating the density of states. As exhibited in Figure 5f, the Fermi level separates the conduction and valence band in the original MnO_2_, and the band gap is 0.69 eV. However, due to the enhancement of the total density state around the Fermi level, the energy gap in Bi-doped MnO_2_ is reduced to 0 eV and a further Fermi level permeates the energy band, indicating that Bi-doped MnO_2_ is a half-metal. Consequently, the conductivity in Bi-doped MnO_2_ was enhanced by the Bi doping. In addition to the influence on ion/electron conductivity and reaction kinetics, the doping of heteroelements also performs a significant role in improving the structural stability of *α*-MnO_2_ during the charge/discharge process. Li et al. [134] adopted the mild hydrothermal method to simultaneously introduce oxygen vacancies and K^+^ into the lattice of *α*-MnO_2_. The synchrotron radiation experiments and DFT calculations show that the insertion of K^+^ not only regulates the type of metal bonds in *α*-MnO_2_ but also changes the average charge distribution of O^2−^. Doping with K^+^ further stabilizes the skeleton structure of *α*-MnO_2_, which prolongs the cycling stability of the cathode. The insertion of extra oxygen vacancies during the K^+^ doping process leads to the formation of *α*-MnO_2_, which can increase the electrochemical reaction active sites and the conductivity of the cathode. Therefore, K-*α*-MnO_2_ exhibits an electrochemical specific capacity up to 250.9 mAh g^−1^ under a current density of 0.2 C and maintains a capacity of 300.2 mAh g^−1^ after 100 cycles. Lan’s group [135] synthesized Cu-doped *α*-MnO_2_ through a mild hydrothermal technique. By comparing the cyclic voltammetry of samples before and after doping in the AZIBs, it was found that the redox peak potential of Cu-doped *α*-MnO_2_ has a smaller offset, indicating that Cu doping can alleviate the polarization of *α*-MnO_2_ and strengthen the structural stability. Liu et al. [133] investigated the electrochemical performance of *α*-MnO_2_ in the process of H^+^/Zn^2+^ intercalation into hydrated ZIBs by replacing Mn^4+^ with transition metals through DFT. Figure 5g,h show the evolution curves of volume change and intercalation state of undoped samples and V- or Cr-doped samples during H^+^ and Zn^2+^ intercalation, respectively. The results illustrate that the substituents contribute to cycling performance and capacity retention. However, as a doping element in *α*-MnO_2_, Cr is more efficient than V in terms of improving discharge voltage, capacity, and cycling stability. The excellent promotion effect of Cr is attributed to the singular atomic and electronic structure of Cr^4+^/Cr^3+^. As an electron acceptor, Cr^4+^ is easier to reduce than Mn^4+^, which hinders the generation of metastable Mn^3+^ and Mn^2+^ centers. Mn^3+^ is less stable than Cr^3+^, the former stabilizing neighboring Mn ions to a large extent.

In addition, the electrochemical property of *α*-MnO_2_ can also be enhanced by doping engineering. For instance, Lu’s team [136] proposed a dual-element-doped *α*-MnO_2_ as a high-performance cathode for AZIBs. In this paper, Ti and Ni co-inserted *α*-MnO_2_ (TiNi-*α*-MnO_2_) was synthesized by a simple hydrothermal strategy in the presence of Ti_3_C_2_X and Ni^2+^, which was employed as a cathode for AZIBs. The insertion of Ti enables multivalent changes (Ti^4+^/Ti^2+^), which are conducive to increasing the specific capacity of the electrode. The lattice distortion caused by Ni can accelerate the Grothus-like proton transfer and enhance the specific capacity of the cathode. Therefore, the TiNi-*α*-MnO_2_ cathode exhibits large reversible capacity and excellent rate capability. Alfaruqi et al. [137] synthesized V-doped *α*-MnO_2_ (V-*α*-MnO_2_) by a simple redox process at room temperature and investigated the electrochemical performance in AZIBs. In the X-ray diffraction pattern, the isotropic displacement of the derivative peak in V-*α*-MnO_2_ relative to pure MnO_2_ illustrates the successful insertion of V into the lattice of MnO_2_. V doping simultaneously increases the specific surface area and electronic conductivity in the MnO_2_ cathode. The Zn^2+^ storage performance test reveals that V-*α*-MnO_2_ exhibits a higher discharge capacity (266 mAh g^−1^) than pure MnO_2_ (213 mAh g^−1^). During the long-term charge/discharge process, the capacity maintenance rate of V-*α*-MnO_2_ (31%) is higher than that of pure MnO_2_, confirming its excellent cycling performance.

In summary, the elemental doping of *α*-MnO_2_ results in the following modification effects: reducing the charge transfer resistance; increasing the ion diffusion rate; promoting the transport of electrons, protons, and Zn^2+^; increasing the electrochemical active sites; alleviating the dissolution of the cathode; and stabilizing the tunnel structure in the cathode. Therefore, it is of great significance to explore the doping engineering of *α*-MnO_2_.

#### 5.1.2. *δ*-MnO_2_

The unique two-dimensional layered structure and large interlayer spacing of *δ*-MnO_2_ are conducive to the intercalation/de-intercalation of H^+^ and Zn^2+^, granting its good specific capacity [62,127]. However, the intrinsic conductivity of *δ*-MnO_2_ is low, and Mn^2+^ dissolution and volume shrinkage are prone to occur in *δ*-MnO_2_-based cathodes during the charging/discharging cycle [138,139,140]. Therefore, it is imperative to modify *δ*-MnO_2_ for its application in AZIBs. Researchers have extensively explored the doping engineering of *δ*-MnO_2_, mainly including cation doping and anion doping. There are many reports on the cation doping modification of *δ*-MnO_2_, containing alkali metals [140], magnetic transition metals [141], another Mg [142] in the third cycle, other Cu [143] and Zn [144] in the fourth cycle, Mo [145] and Ag [146] in the fifth cycle, and Ce [51] and Bi [143] in the sixth cycle. There are a few reports on anion doping modification, including F [147] and S [35].

**Figure 6:**
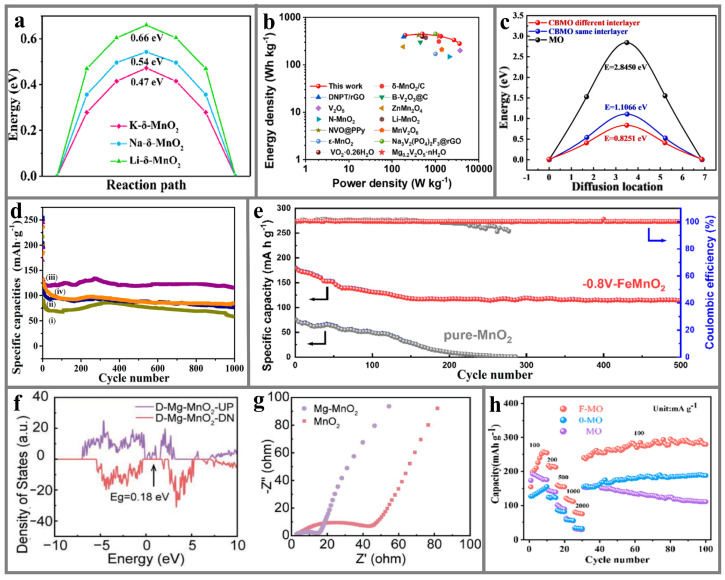
(**a**) Zn^2+^ migration barriers of Li-, Na-, and K-*δ*-MnO_2_ [140]. Copyright 2021, Elsevier. (**b**) Performance comparison of CS@Ce-MnO_2_-15 electrode and other cathodes (the mass ratio of KMnO_4_/CS was 15:1) [51]. Copyright 2023, Elsevier. (**c**) Comparison of energy barriers of Zn^2+^ transfer in *δ*-MnO_2_ and Cu/Bi-doped *δ*-MnO_2_ [148]. Copyright 2023, Elsevier. (**d**) Cycling performance of (i) *δ*-MnO_2_, (ii) *δ*-MnO_2_-1 (the molar ratio of AgNO_3_/KMnO_4_ was 1:100), (iii) *δ*-MnO_2_-2 (the molar ratio of AgNO_3_/KMnO_4_ was 2:100), and (iv) *δ*-MnO_2_-3 (the molar ratio of AgNO_3_/KMnO_4_ was 10:100) [146]. Copyright © 2023, American Chemical Society. (**e**) Cycling stability test of Fe-doped *δ*-MnO_2_ [141]. Copyright 2023, Elsevier. (**f**) Density of Mg^2+^-doped δ-MnO_2_ with accompanying oxygen vacancy [142]. (**g**) Nyquist plots of Mg–*δ*-MnO_2_ and *δ*-MnO_2_ [142]. Copyright © 2023, Wiley. (**h**) Rate performance in *δ*-MnO_2_ through air quenching (MO), *δ*-MnO_2_ through water quenching (0-MO), and *δ*-MnO_2_ through quenching in aqueous potassium fluoride solution (FMO) [147]. Copyright 2023, Elsevier.

Qi et al. [140] used a simple room-temperature redox method to pre-intercalate alkali metals including Li^+^, Na^+^, and K^+^ into the interlayer of MnO_2_, which was utilized as a cathode in AZIBs, and explored their different effects on the electrochemical property and energy storage mechanism in AZIBs. The electrochemical test results indicate that the specific capacity, rate capability, and cycling stability of the cathode are proportional to the radius of the alkali metal. The larger the radius of the doping ions, the larger the interlayer spacing of the cathodes and the easier the diffusion of Zn^2+^. The Zn^2+^ diffusion barriers of Li-, Na-, and K-*δ*-MnO_2_ obtained from DFT are 0.7, 0.5, and 0.5 eV (Figure 6a), respectively, indicating that K-*δ*-MnO_2_ is able to contribute more excellent electrochemical kinetics. Yu et al. [51] synthesized a Ce-*δ*-MnO_2_@CS composite composed of a mesoporous carbon core and Ce-doped MnO_2_ nanosheet shell by a simple low-temperature liquid-phase reaction method. Ce serves as a pillar to expand the interlayer spacing of MnO_2_, which can promote the intercalation/de-intercalation of Zn^2+^. The network structure of mesoporous carbon spheres and unique core–shell structure offers an effective pathway for the conduction of ions and electrons. The optimized Ce-*δ*-MnO_2_@CS electrode demonstrates significantly improved energy density and power density (Figure 6b). With the mechanical flexibility, the Ce-*δ*-MnO_2_@CS core–shell composite is expected to be applied in wearable flexible electronic devices. Li et al. [148] pre-embedded Cu and Bi double transition metal ions between *δ*-MnO_2_ layers (CuBi-*δ*-MnO_2_) through a one-step hydrothermal strategy, which served as a cathode for AZIBs. They also used a DFT calculation method to compare the diffusion barriers of Zn^2+^ in MO and CuBi-*δ*-MnO_2_. The formation energy of origin Zn^2+^ transfer paths between the same layer and different layers in CuBi-*δ*-MnO_2_ are −4.71 and −5.94 eV, respectively. The diffusion barriers of Zn^2+^ in CuBi-*δ*-MnO_2_ are 1.1066 and 0.8251 eV when it is in the same layer and different layer with Cu/Bi ions, respectively (Figure 6c). Both of them are lower than in *δ*-MnO_2_ (2.845 eV). The impurity doping can introduce numerous defects in δ-MnO_2_ and embed more Zn^2+^ as an active site. Wu’s group [146] employed a mild one-step hydrothermal method to generate Ag-inserted *δ*-MnO_2_ composites (Ag-*δ*-MnO_2_) with different concentrations as cathodes of AZIBs. The doping of Ag^+^ should introduce abundant oxygen defects and act as an active storage site for Zn^2+^, which facilitates Zn^2+^ migration. Electrochemical characterization reveals that when the doping concentration is 1.12 wt %, Ag-*δ*-MnO_2_ exhibits the best specific capacity and cycling stability. After 1000 cycles, the discharge capacity can still retain 114 mAh g^−1^ (Figure 6d). Yan et al. [141] anchored Fe^3+^, Co^2+^, and Ni^2+^ ions between the layers of *δ*-MnO_2_ (Fe-, Co-, Ni-*δ*-MnO_2_) by a dual-field in situ induction process, which selectively accelerated the transport of either H^+^ or Zn^2+^. The study discovered that the insertion of Fe^3+^ preferentially promotes the transfer of Zn^2+^, and the insertion of Co^2+^ and Ni^2+^ preferentially promotes the transfer of H^+^. The doping of magnetic transition metal ions into the oxygen vacancies of *δ*-MnO_2_ can buffer the structural change caused by lattice distortion during the charging/discharging process, increase the active storage sites of H^+^ and Zn^2+^, and accelerate the ions’ diffusion. The results show that Fe-*δ*-MnO_2_ still exhibits a high capacity of more than 110 mAh g^−1^ after 500 cycles. However, the discharge capacity of pure *δ*-MnO_2_ was reduced to less than 25 mAh g^−1^ after 250 cycles (Figure 6e). Sun’s group [35] prepared S-doped *δ*-MnO_2_ by a low-temperature vulcanization method and investigated its zinc storage performance. It was found that S-doped *δ*-MnO_2_ had a higher discharge specific capacity and pseudocapacitance contribution rate than pure *δ*-MnO_2_. They believe that this is due to the oxygen defects generated by S doping on the amorphous surface of *δ*-MnO_2_, improving the Zn^2+^ storage site, and this special amorphous region gives ions the ability to enter the electrolyte/electrode interface and contribute to the capacitance.

The defects introduced by this special strategy can not only provide abundant active sites to insert Zn^2+^ but can also enhance the ionic/electronic conductivity of *δ*-MnO_2_. Di’s group [142] synthesized Mg^2+^-doped *δ*-MnO_2_ (Mg-*δ*-MnO_2_) via a mild hydrothermal process and employed it as a cathode for AZIBs. Oxygen vacancies are also introduced during the Mg^2+^ doping, which significantly improves the electronic conductivity and ion diffusion coefficient of *δ*-MnO_2_. In particular, they showed that Mg-doped *δ*-MnO_2_ with oxygen vacancies displays the lowest band gap (0.18 eV, Figure 6f) by DFT calculations. At the same time, new electronic states appear around the Fermi level, indicating that the inherent electron transfer efficiency and electrochemical reactivity have been improved. The results obtained from electrochemical impedance spectroscopy indicate that the charge transfer resistance of Mg-MnO_2_ is lower than that of MnO_2_ (Figure 6g). Besides the effect of the introduced oxygen vacancies, the band gap change caused by doping can also enhance its electronic/ionic conductivity. Ding’s team [149] calculated the density of states of *δ*-MnO_2_ and Cr_0.02_Mn_0.98_O_2_ by DFT and found that, compared with *δ*-MnO_2_, the introduction of Cr causes the density of states in *δ*-MnO_2_ to move toward the Fermi level, illustrating that the presence of Cr enlarges the dedication of spin-down electrons to the density of states around the Fermi level, resulting in a band gap decrease. Therefore, the doping of Cr can efficiently accelerate the electron immigration of *δ*-MnO_2_, further increasing the conductivity of the electrode.

The doping of other metals can also adjust the ionic bonding properties of *δ*-MnO_2_, thereby improving the structural stability of *δ*-MnO_2_ and achieving a longer service life in AZIBs. Wang et al. [147] synthesized highly oriented F-doped *δ*-MnO_2_ nanosheets (F-*δ*-MnO_2_) as a cathode for AZIBs by the lava method combined with annealing post-treatment. F doping can not only stabilized the [MnO_6_] octahedral structure by forming F-Mn chemical bonds but also ensured the structural stability of *δ*-MnO_2_. The charge compensation effect caused by F doping was verified by the XPS test, that is, F doping can enhance the Mn^3+^ concentration. Therefore, the dissolution of Mn active substances was inhibited by adjusting the proportion of Mn^3+^/Mn^4+^ through F doping. F-doped *δ*-MnO_2_ exhibits excellent rate performance, with discharge capacities of 288, 240, 160, 122, and 84 mAh g^−1^ under current densities of 100, 200, 500, 1000, and 2000 mA g^−1^, respectively. When the current density was restored to 100 mA g^−1^ again, the F-doped *δ*-MnO_2_ still maintained a high capacity of 280 mAh g^−1^ (Figure 6h). These results demonstrate that doping engineering can promote the structural distortion of the [MnO_6_] octahedron, improve the reversibility of the electrochemical reaction, and thus maintain the long-term cycling stability of the material structure. Sun’s group [150] investigated the impact of doping elements on the cycling stability of Cr and Ni co-doped *δ*-MnO_2_ through comprehensive structural and performance characterization. The results show that Cr^4+^ aggravates the Jahn–Teller distortion of Mn(III)O_6_ and promotes the dissolution of CrNi-ZnMn_2_O_4_ into Mn^2+^. The doped Cr^3+^ can be used as ‘scissors’ to eliminate the low activity MnO_2_ accumulated by the disproportionation of dissolved Mn^3+^. Therefore, the Cr and Ni elements enable the CrNi-MnO_2_ to undergo a highly reversible MnO_2_/Mn^2+^ redox reaction and maintain the structural integrity after long-term cycling stability testing. In subsequent work by the same research group, Mo was found to play a similar role in Mo-doped *δ*-MnO_2_ [145].

In summary, the doping modification of *δ*-MnO_2_ has the following improvement effects: the doping of metal or non-metal elements will simultaneously produce oxygen vacancies and expand the interlayer spacing of *δ*-MnO_2_, and both the doped atom and vacancy can simultaneously increase the electron mobility of the bulk material while reducing the ion diffusion barrier, thus promoting the reaction kinetics of the cathode; moreover, doping modification can buffer the structural change caused by lattice distortion in the charge/discharge process, then strengthen the cycling stability while maintaining the stability of the skeleton structure. Therefore, it is of great importance to explore the doping engineering of *δ*-MnO_2_.

#### 5.1.3. *β*-MnO_2_, *ε*-MnO_2_, and *γ*-MnO_2_

Although the open channels of *β*-MnO_2_ with thermodynamic stability [122] can accommodate a reasonable amount of Zn^2+^, the small size of the channels hinders the ion diffusion during the cycling process, resulting in the slow reaction kinetics of the *β*-MnO_2_ cathode. The narrow tunnel structure of *β*-MnO_2_ reduces the active sites for electrochemical reactions, resulting in the low specific capacity of the *β*-MnO_2_ cathode [151,152]. In order to solve these challenges and apply *β*-MnO_2_ to the cathode in AZIBs, a large number of papers on doped *β*-MnO_2_ have been reported in recent years. One group synthesized Eu-*β*-MnO_2_ by doping rare earth element Eu into *β*-MnO_2_ through a hydrothermal process and used it as a cathode in AZIBs. Eu has good conductivity and stable chemical properties, which is one of the best choices for the doping modification of *β*-MnO_2_. It is found that the intercalation of Eu enlarges the interlayer spacing of *β*-MnO_2_, promotes the diffusion of H^+^ and Zn^2+^, and maintains the structural stability of *β*-MnO_2_. Eu-β-Mn O2 has a high specific capacity at low current density and can still display a discharge specific capacity of 254 mAh g^−1^ after 128 cycles. Doping manganese oxide with rare earth elements is one of the research hotspots in the modification of AZIB cathode materials [153].

The dense and limited three-dimensional tunnel structure in *ε*-MnO_2_ hinders the intercalation/de-intercalation of protons and cations, which leads to the low conductivity of the *ε*-MnO_2_ cathode [154]. As an effective strategy to improve the electrochemical activity of electrode materials, it is of great importance to explore the doping engineering of *ε*-MnO_2_. Zhang et al. synthesized Cu^2+^-doped *ε*-MnO_2_ porous nanostructures (Cu-*ε*-MnO_2_) by a simple one-step electrodeposition process for an AZIB cathode. The insertion of Cu^2+^ increases the spacing of the *δ*-MnO_2_ tunnel structure and increases the diffusion rate of electrons and ions. The micropores promote charge storage and ion adsorption, and the mesopores provide ion transport channels. Cu-*ε*-MnO_2_ has better electrochemical performance than pure MnO_2_, and under a current density of 0.2 A g^−1^, the discharge specific capacity can reach 235 mAh g^−1^ [155].

The multi-tunnel structure of *γ*-MnO_2_ is conducive to the intercalation/de-intercalation of cations, and the formed porous structure can offer abundant active sites for electrochemical reactions [58]. However, the irregular arrangement of *γ*-MnO_2_ crystal cells leads to low crystallinity, which contributes to the uneven distribution of potential and irreversible phase transition during the charging/discharging process. Hence, it is very important to change the crystal structure of *γ*-MnO_2_ by doping modification to improve its Zn^2+^ storage performance. Wang’s group synthesized a Ni^2+^-doped *γ*-MnO_2_ (Ni-*γ*-MnO_2_) as a highly active cathode material for AZIBs by employing a mild one-step electrodeposition approach [156]. It was found that the doping of Ni^2+^ reduces the diffusion barrier of protons, which is beneficial for the insertion of ions into the tunnel structure and accelerates the reaction kinetics of the battery. DFT calculations show that the insertion of Ni^2+^ improves the electronic conductivity between [MnO_6_] octahedra. Therefore, the Ni-*γ*-MnO_2_ cathode exhibits excellent rate performance (56 mAh g^−1^ at a current density of 10 A g^−1^) and a long cycle life (more than 100% capacity retention after 11,000 cycles at 3.0 A g^−1^).

### 5.2. MnO

As the simplest oxide among Mn_x_O_y_-based cathode materials, the storage mechanism of MnO involves the reversible co-intercalation/de-intercalation of H^+^ and Zn^2+^ and their chemical conversion. Although a higher theoretical capacity, higher conversion voltage, and higher energy density make MnO more competitive, it also has limitations such as fewer active sites, poor electronic conductivity, and poor cycling performance. This section primarily focuses on the impact of MnO doping engineering on enhancing the electrochemical performance of AZIBs. There are several effective strategies for MnO doping engineering: co-doping to boost intrinsic conductivity, high-entropy doping to improve structural stability, introducing vacancies to increase active sites, inducing changes in the morphology and structure of MnO and enriching the porosity, and mitigating manganese dissolution to enhance electrochemical performance. Therefore, doping engineering has been widely employed to address the aforementioned deficiencies of MnO, thereby improving its practical applicability.

**Figure 7:**
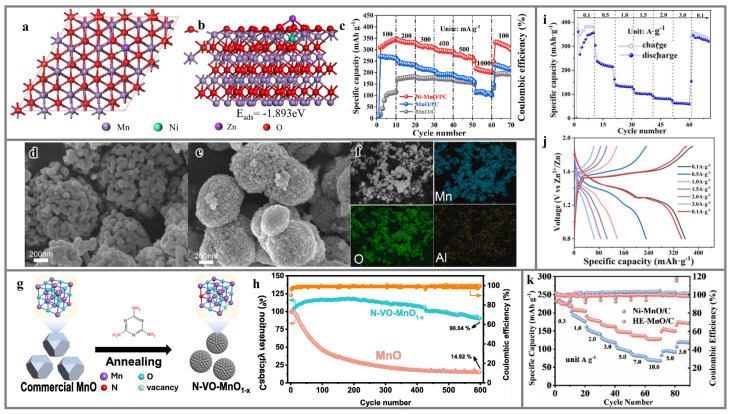
(**a**) The adsorption structure models of Ni-MnO. (**b**) The adsorption energy for Zn^2+^ of perfect Ni-MnO. (**c**) Rate performance of Ni-MnO/porous carbon, MnO/porous carbon, and MnO/carbon [157]. Copyright 2023, Elsevier. SEM images of pure MnO (**d**) and 5% Al-MnO (**e**) and the corresponding elemental mapping of 5% Al–MnO (**f**) [53]. Copyright 2022, Elsevier. (**g**) Schematic illustration of fabrication of N-doped MnO_1−x_/oxygen vacancy through facile one-step melamine pyrolysis technology. (**h**) Long-term cycling stability at a current density of 0.5 A g^−1^ of N-doped MnO_1−x_/oxygen vacancy and MnO [158]. Copyright 2021, Elsevier. (**i**) Rate performance of ZnCo-MnO/C. (**j**) Galvanostatic charge/discharge profiles at different current densities of ZnCo-MnO/C [160]. Copyright 2022, Elsevier. (**k**) Rate performance of high-entropy-doped MnO/C and Ni-MnO/C [161]. Copyright 2024, Elsevier.

To enhance the Zn^2+^ storage capacity of the MnO cathode, Cao’s group [157] synthesized Ni-nanoparticle-doped MnO composites (Ni-MnO/PC) that were uniformly anchored on porous carbon through hydrothermal and annealing methods. As shown in Figure 7a,b, Ni-MnO exhibits a stronger Zn^2+^ adsorption energy, suggesting a higher affinity for Zn^2+^ adsorption. The incorporation of porous carbon provides an abundance of pores and ensures sufficient contact between the cathode and the electrolyte, providing sufficient diffusion pathways for ions. Furthermore, the addition of Ni nanoparticles promotes electron rearrangement, which in turn improves the conductivity of nanomaterials. As shown in Figure 7c, the Zn||Ni-MnO/PC battery exhibits a discharge specific capacity of 347.4 mAh g^−1^ at a current density of 100 mA g^−1^. Even at a higher current density (3000 mA g^−1^), the Ni-doped MnO/PC electrode still maintains a capacity retention rate of more than 90% after long-term cycling, which is superior to MnO/PC and MnO/C. In addition, the team also verified that the introduction of vacancies can effectively increase the distribution of active sites and further improve the electrochemical performance of MnO cathode materials. Chen’s group [53] introduced a strategy of Al doping to modify MnO, resulting in the synthesis of Al-MnO materials. These materials can be transformed into orthorhombic manganese ore-structured MnO_2_ (*R*-MnO_2_) through co-precipitation and calcination processes. Through scanning electron microscope (SEM) images, it can be found that MnO/Al-MnO forms microspheres with diameters in the range of approximately 0.6~0.8 μm (Figure 7d,e), and Al is uniformly distributed throughout the sample (Figure 7f). This Al^3+^ doping not only introduces an abundance of Mn vacancies but also increases the specific surface area and pore size of MnO. This enhancement improves the cathode’s wettability with the electrolyte. Furthermore, it can also reduce the ion transport path within the crystal structure and provide more active sites. Similarly, Liang’s group [158] synthesized N-doped MnO through a one-step melamine pyrolysis method. This process introduced oxygen vacancies into the material (Figure 7g). Oxygen vacancies significantly enhance the material’s intrinsic electronic conductivity and increase the distribution of electrochemically active sites for Zn^2+^ storage. On the other hand, they can also promote the insertion and extraction of Zn^2+^, thus greatly improving the electrochemical performance of the inert MnO. The fabricated N-VO-MnO_1−x_ cathode demonstrates excellent rate performance (after 600 cycles at 0.5 A g^−1^, there is still a retention rate of 90%, Figure 7h). Lei’s group [159] doped trace amounts of calcium into manganese monoxide (CMO) using a solid-state reaction, creating a cathode material of AZIBs with rich interfacial chemical bonds. In addition, calcium doping not only optimizes the charge/ion state and electronic band gap but also ensures a reversible phase transition and mitigates the dissolution of Mn from the cathode. Concurrently, the wide lattice spacing of the CMO material not only weakens the interaction force between anions and cations but also provides more space channels for ion migration during the initial cycle, significantly enhancing the diffusion kinetics.

Since the MnO cathode material modified by single-ion doping has not yet met expectations, a plethora of research has focused on the advancement of multi-ion doping strategies for the enhancement of MnO cathode materials. Distinct from the conventional single-ion doping approach, Zn/Co co-doped MnO/C was prepared by Chen’s group [160] using metal–organic frameworks as precursors and used as an AZIB cathode material. The doping of Zn^2+^ enhances the reactivity of MnO, while the incorporation of Co ions boosts the capacity. Moreover, Co ions can also inhibit the Jahn–Teller effect of Mn^3+^ in the electrolyte, thereby enhancing structural stability. Benefiting from the synergistic effect of the two doped ions, the ion diffusion rate and conductivity of MnO are remarkably enhanced, thus exhibiting excellent electrochemical performance (Figure 7i,j). Unlike single-ion doping, multi-ion doping realizes the in situ bonding of manganese through the close arrangement of different heteroatoms, leading to the formation of robust manganese ion bindings within the crystal cell. Recently, Wang’s group [161] prepared Co, Fe, Ni, Cu, and Cr co-doped MnO cathode materials (co-doped MnO) by a high-entropy-doping strategy. The molar contents of the five heteroelements is similar, and they all have the same molar ratio with Mn ions (Mn:X = 28.3:1). The interactions between the metal elements in the co-doped MnO promote a denser overlap of the electron cloud between Mn^2+^ and O^2−^, which greatly increases the binding energy of the MnO bond. In addition, a large number of oxygen defects introduced by Co, Fe, Ni, Cu, and Cr doping can accelerate the ion transport in the cathode material and enhance the reaction kinetics. Finally, this co-doped high-entropy MnO exhibits excellent long-term cycle stability and rate performance (Figure 7k).

In summary, to address these issues (fewer active sites, poor electronic conductivity, and poor cycling performance) with MnO cathode materials, the prevailing strategies include co-doping to enhance intrinsic conductivity and high-entropy doping to bolster structural stability. Through doping engineering, a protective layer is introduced on the surface of MnO, which activates the inert phase, accelerates diffusion kinetics, boosts electronic conductivity, and mitigates manganese dissolution, thereby improving electrochemical performance. Additionally, doping engineering can introduce vacancies or defects to enhance the diffusion performance of Zn^2+^ within the batteries.

### 5.3. Mn_2_O_3_

**Figure 8:**
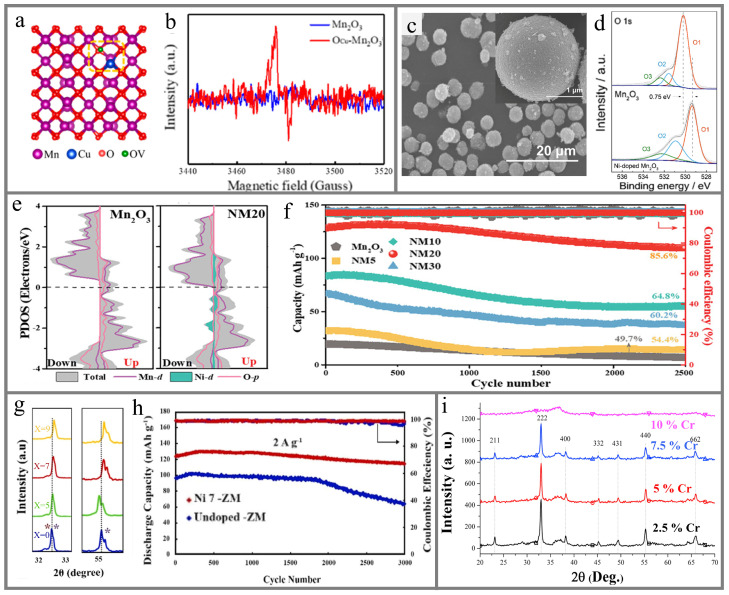
(**a**) SEM images of Ni-doped Mn_2_O_3_ [162]. (**b**) Electron paramagnetic resonance spectroscopy of Ocu-Mn_2_O_3_ and Mn_2_O_3_ [164]. Copyright © 2023, American Chemical Society. (**c**) Crystalline diagram of Ocu-Mn_2_O_3_ [54]. (**d**) High-resolution XPS spectra of O 1s for Mn_2_O_3_ and Ni-doped Mn_2_O_3_ [54]. Copyright 2022, Elsevier. (**e**)The projected density of states (PDOS) of the Mn_2_O_3_ and Ni-doped Mn_2_O_3_. (**f**) Cycling performance of Ni_7_-ZnMn_2_O_4_/Mn_2_O_3_ and undoped ZnMn_2_O_4_/Mn_2_O_3_ electrodes at a current density of 2 A g^−1^ [28]. Copyright © 2021 Wiley. (**g**) XRD pattern of Nix-ZnMn_2_O_4_/Mn_2_O_3_ with various Ni^2+^ contents (x = 0, 5, 7, and 9); enlarged pattern of dislocated peaks of the XRD. The asterisk on the left represents the characteristic peak of Mn_2_O_3_, and the one on the right represents ZnMn_2_O_4_ [165]. (**h**) Cycle performances of Mn_2_O_3_, Ni-doped Mn_2_O_3_, -10, -20, and -30 [166]. Copyright 2023, Elsevier. (**i**) X-ray diffraction patterns of the samples synthesized from manganese nitrate solutions containing 2.5%, 5%, 7.5%, and 10% Cr^6+^ [167]. Copyright 2017, Elsevier.

Although Mn_2_O_3_ has the superiority of high energy density and low production cost, it has the worst electrostatic instability compared with other crystalline phases of Mn_x_O_y_ materials because the outermost 3d^4^ electron configuration of trivalent manganese ions is more prone to electron transfer. However, this electrostatic instability leads to the reduction or oxidation of Mn_2_O_3_ during the electrode reaction, thus destroying its chemical morphology and structure. To improve the electrostatic stability of Mn_2_O_3_, Zhang et al. [162] designed an oxygen-deficient Mn_2_O_3_ cathode by doping with positive monovalent Cu ions (Figure 8a). They confirmed the presence of oxygen defects within the material using electron paramagnetic resonance spectroscopy (Figure 8b). These oxygen defects are instrumental in modifying the internal electric field of the material by compensating for the non-zero dipole moment, which in turn significantly enhances the material’s electrostatic stability. Furthermore, the Cu-doped Mn_2_O_3_ electrode demonstrates a substantial diffusion coefficient and commendable rate performance, ranging from 1 × 10^−6^ to 1 × 10^−8^, coupled with a high degree of reversible cycling stability. At the same time, the construction of stronger ionic bonds by metal ion doping is also one of the effective methods to increase the stability of materials. Baeck et al. [54] successfully synthesized Ni-doped Mn_2_O_3_ microspheres with excellent electrochemical properties through co-precipitation and subsequent heat treatment (Figure 8c). On the other hand, the introduction of Ni makes a large number of Ni-O-Mn interfaces appear in Mn_2_O_3_, in which the electronic structure of Ni-doped Mn_2_O_3_ is well designed by effectively optimizing the adsorption energy of the intermediate. To confirm this, the XPS spectra of O 1s for Mn_2_O_3_ and Ni-doped Mn_2_O_3_ are examined and shown in Figure 8d. In order to eliminate the influence of surface contamination caused by carbon and oxygen pollutants in the atmosphere, Ar^+^ ion beam sputtering was employed prior to the XPS test [163]. The O 1s spectrum of Ni-doped Mn_2_O_3_ can be divided into three peaks located at 529.4 eV, 530.9 eV, and 532.2 eV; the binding energy is different from the O adsorbed on the surface of the solid material (531.5 eV), so the three peaks are attributed to the metal–oxygen bond (O1), the O atom (O2) in the hydroxyl group, and the surface O defect site (O3), respectively [164]. Compared to pure Mn_2_O_3_, the O 1s binding energy of Ni-doped Mn_2_O_3_ is negatively shifted by 0.75 eV. This change is mainly due to the doping of Ni into Mn_2_O_3_, which enhances the ionic bond in Mn_2_O_3_ to a certain extent, thereby enhancing the stability of the material. On the one hand, its excellent performance is due to the fact that the hierarchical and rough surface structure provides a larger active surface area and abundant active sites (Figure 8d), thereby achieving efficient mass transfer. In addition, Huang et al. [28] also found that the doping of the Ni element can effectively alleviate the dissolution of Mn^3+^ in Mn_2_O_3_. As shown in Figure 8e, the incorporation of Ni^2+^ increases the conductivity of Mn_2_O_3_ due to the slight differences around the Fermi level. Furthermore, the presence of Ni^2+^ facilitates electron rearrangement, which enhances the overall conductivity and ultimately improves the reaction kinetics and the electrochemical performance of Ni-Mn_2_O_3_. The intercalation of Ni^2+^ into the crystal lattice of Mn_2_O_3_ reduces its overall formation energy, thereby effectively enhancing the stability of the material and mitigating the dissolution of Mn. Consequently, the resulting NM cathode exhibits a higher specific capacity and a longer life (Figure 8f).

Doping engineering can also increase the distribution of active sites in Mn_2_O_3_ materials or enhance the reaction kinetics to optimize the performance of the materials. Javanbakht’s group [165] synthesized Ni-doped ZnMn_2_O_4_/Mn_2_O_3_ nanocomposites via pulse potential electrodeposition which were subsequently used as cathode materials for AZIBs. By analyzing the binding energy of the surface elements of the Ni-doped ZnMn_2_O_4_/Mn_2_O_3_ nanocomposites (Figure 8g), it can be seen that the characteristic peaks of Mn_2_O_3_ gradually shift with increasing Ni content. These changes are primarily attributed to the incorporation of Ni^2+^, which realizes the effective regulation of Mn^3+^ and Mn^4+^ concentrations (the concentration of Mn^3+^ decreases and that of Mn^4+^ increases). In addition, the incorporation of Ni^2+^ also reduces the potential gap and improves the reversible insertion/extraction of Zn^2+^ in the Ni-doped ZnMn_2_O_4_/Mn_2_O_3_. The Ni-doped ZnMn_2_O_4_/Mn_2_O_3_ nanocomposites still exhibit a discharge capacity of 114.67 m Ah g^−1^ after a long-term cycle stability test (at 2 A g^−1^ after 3000 cycles), which is much higher than that of the undoped nanocomposites. Davarani et al. [167] proposed a strategy to prepare Cr-doped Mn_2_O_3_ with cauliflower-like nanostructures through constant-current cathodic electrodeposition. In this process, a Mn^2+^ nitrate solution containing a small amount of dichromate was used as the raw material. During the synthesis process, the dispersed Cr ions in the solution played a certain role in inducing the preferential formation of MnO_2_ and then reacted with excess Mn^2+^ to form Mn_2_O_3_ nanostructures. The introduction of Cr reduces the crystallinity and improves the morphology of Mn_2_O_3_ products (Figure 8i) and finally shows superior performance compared to undoped manganese oxide materials. Ravi et al. [166] have developed self-assembled, three-dimensional, mesoporous, original *α*-Mn_2_O_3_ microspheres, as well as neodymium (Nd)-doped variants, using a simple hydrothermal method. With 5% Nd doping, the Nd-Mn_2_O_3_ exhibits a uniform morphological structure and an increased number of oxygen vacancies. These characteristics not only make the material distribute more electrochemical active sites but also shorten the diffusion distance of ions in the Mn_2_O_3_ cathode material. As a result, the Mn_2_O_3_ electrode demonstrates outstanding electrochemical activity, abundant ion mobility, a high specific capacity, and long cycle stability.

### 5.4. Mn_3_O_4_

**Figure 9:**
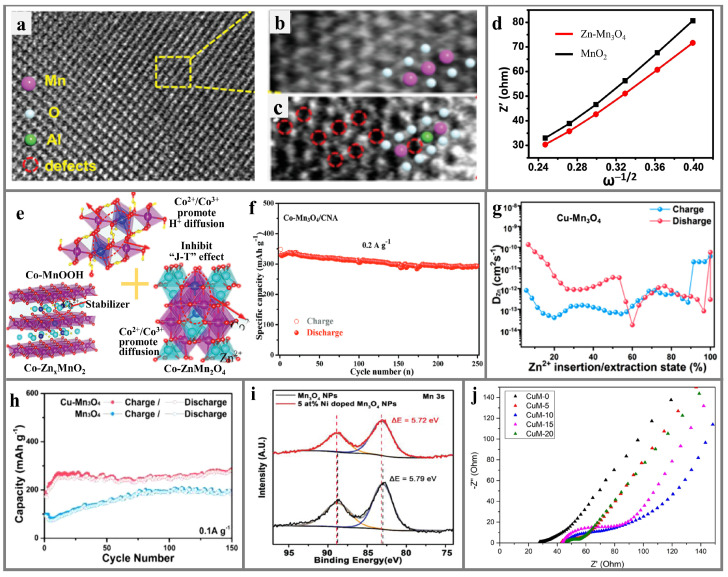
(**a**) Aberration-corrected TEM images of Mn_3_O_4_. HAADF-STEM images of Mn_3_O_4_ (**b**) and Al_0.48_Mn_2.52_O_4_ (**c**) [168]. Copyright © 2021 Wiley. (**d**) The relationships between Z’re and ω^1/2^ for Zn-doped Mn_3_O_4_ nanosheets and MnO_2_ nanosheets [52]. Copyright © 2022 Wiley. (**e**) Structure diagram of intermediate products. Mn, Co, O, Zn, and H elements are represented by purple, blue, red, blue, and yellow spheres, respectively. (**f**) The cycle performance diagram at low current density (at 0.2 A g^−1^) of the Co-doped Mn_3_O_4_/carbon nanosheet array [170]. Copyright © 2020 Wiley. (**g**) Zn^2+^ diffusion coefficient of Cu-doped Mn_3_O_4_. (**h**) Cycling stability of Cu-doped Mn_3_O_4_ and Mn_3_O_4_ at 100 mA g^−1^ [171]. Copyright 2021, Elsevier. (**i**) High-resolution XPS spectra of Mn 3s for Mn_3_O_4_ and Ni-doped Mn_3_O_4_. Copyright © 2020 Wiley. (**j**) EIS curves of Mn_3_O_4_ films with different copper doping amounts [175]. Copyright © 2023 American Chemical Society.

As a typical spinel metal oxide, Mn_3_O_4_ is considered to be one of the cathode materials with great research significance for AZIBs due to its unique electronic structure, mixed-valence Mn^2+^/^3+^ center, and unique three-dimensional pore structure [168,169]. However, the rapid capacity fade and poor rate performance hinder its commercial application. It is generally known that improving the diffusion kinetics of materials during charge and discharge is crucial to optimize the rate performance of electrode materials. Shi et al. [169] synthesized mesoporous Al_0.35_Mn_2.52_O_4_ with an enhanced specific surface area through a selective leaching process that targets the removal of aluminum (Al). As shown in Figure 9a–c, the spinel structure of Mn_3_O_4_ is endowed with a multitude of defects by removing about 30% of Al ions. Characterization studies reveal that Zn^2+^ has a faster diffusion rate in Al_0.35_Mn_2.52_O_4_ with rich Mn vacancies. Concurrently, the absence of a significant electrostatic barrier, coupled with the heightened mobility of Zn^2+^, results in accelerated electrode kinetics. Furthermore, since H^+^ tends to adsorb on the oxygen bridge site during the migration process and is also electrostatically repelled by the adjacent Mn in Mn_3_O_4_, the vacancy defect is also beneficial to reduce the diffusion barrier of H^+^. Lin et al. [52] synthesized Zn-doped Mn_3_O_4_ and *γ*-MnO_2_ nanocomposites (ZnMM-NSs) using an electrochemical deposition method. This process was conducted directly on the surface of nickel foam that had been modified with silver nanoparticles and carbon nanotubes, resulting in a vertically oriented three-dimensional porous nanosheet framework. It was observed that the doping of zinc ions creates an expedited path for both electron and ion diffusion. Compared with the undoped MnO_2_-NSs, the ZnMM-NSs electrode exhibited a larger Warburg slope at low frequencies (Figure 9d), indicating that it has faster diffusion kinetics.

It is worth noting that the enhancement of the intrinsic electronic/ionic conductivity of the material is very important for the optimization of the rate performance of the Mn_3_O_4_ materials. Fortunately, doping engineering has been proved to be a meaningful way to improve the electronic/ionic conductivity of Mn_3_O_4_ materials. Wang et al. [170] have reported on a multivalent cobalt-doped Mn_3_O_4_ with high capacity and reversibility and have investigated the roles of cobalt ions with different valences. Among them, Co^2+^ serves as a ‘structural pillar’ between the layers of intermediates (*δ*-MnO_2_) of the cycle, while Co^4+^ within the layer enhances the conductivity of Mn^4+^ and helps to maintain a high specific capacity. Most notably, the introduction of Co^2+^ and Co^3+^ into the Mn_3_O_4_ structure can effectively alleviate the Jahn–Teller effect of Mn^3+^ during the cycling process and significantly guarantee the stability of the material structure (Figure 9e). The resulting Co-Mn_3_O_4_ cathode still maintains a substantial discharge specific capacity of 292.6 mAh g^−1^ after 250 cycles at 200 mA g^−1^, with a commendable capacity retention rate of 90% (Figure 9f). Kong et al. [171] developed Cu-doped Mn_3_O_4_ as a cathode material for AZIBs. Due to the strong affinity, Cu^2+^ partially substitutes for Mn^3+^ within the manganese oxide lattice, culminating in the formation of a porous micro/nanostructure consisting of numerous irregular nanoparticles. As shown in Figure 9g, the conductivity and Zn^2+^ diffusivity of the Cu-Mn_3_O_4_ electrode is significantly enhanced by the Cu^2+^ doping. The Cu-Mn_3_O_4_ cathode achieves a discharge capacity of 250 mAh g^−1^ under 100 mA g^−1^, surpassing that of the pure Mn_3_O_4_ electrode (150 mAh g^−1^) (Figure 9h). In addition, the doping of other metal elements can also enhance the conductivity of the material by changing the element arrangement of the contact surface between the material and the electrolyte. For instance, Li et al. [172] synthesized cobalt-doped manganese oxide nanoparticles. Structural and electrochemical performance characterization demonstrated that Co doping enhanced the conductivity of [MnO_6_] octahedra and facilitated the electron transport of Co-Mn_3_O_4_ during both charging and discharging processes. Additionally, Co doping enhanced the diffusion of Zn^2+^ on the surface of ZnMn_2_O_4_ at the AC anode.

Doping engineering can also enhance the chemical activity of Mn_3_O_4_ materials by adding more electrochemically active sites. Based on the study of electrodynamics, Nam et al. [173] discovered that Ni-doped Mn_3_O_4_ with a doping level of 5% also exhibits enhanced electrochemical activity. This is mainly attributed to the distortion of the crystal structure of Mn_3_O_4_ nanoparticles induced by the Ni doping. Such lattice distortion results in localized strain, which in turn manipulates the electronic structure (Figure 9i) and potentially increases the number of the electrochemical active sites. Zhang’s group [174] uniformly dispersed the Fe element in ultrathin Mn_3_O_4_ nanosheets. They discovered that Fe doping not only increases the distribution of electrochemical active sites but also confers excellent electrochemical activity to Fe-Mn_3_O_4_ by adjusting the d-band center of Mn_3_O_4_ and modifying the adsorption energy of oxygen-containing intermediates. On the other hand, the introduction of heteroatoms can induce the controllable evolution of the morphology and structure of the material, which in turn affects the content of electrochemical active sites. Yeenduguli et al. [175] prepared Cu-doped Mn_3_O_4_ thin films by using the spray pyrolysis technique and conducted a detailed study of their structure and electrochemical properties. The addition of Cu not only changed the surface morphology and roughness but also significantly affected the overall morphology of Mn_3_O_4_. Furthermore, atomic force microscopy results revealed that when the Cu content reached 10 at%, the surface of the film became smooth, but the roughness paradoxically increased. This modification also led to an increased distribution of electrochemical active sites. Analysis of electrochemical impedance spectroscopy data revealed that the Cu-doped Mn_3_O_4_ film can significantly reduce the electron transfer impedance of the film (Figure 9j).

## 6. Conclusions and Perspective

This review focuses on doped Mn_x_O_y_ cathodes in AZIBs. First, the structural characteristics of Mn_x_O_y_ with different oxidation states and crystal phases, the Zn^2+^ storage mechanisms of Mn_x_O_y_-based AZIBs, and the problems and optimization strategies of doped Mn_x_O_y_ cathodes are briefly introduced. Then, the electrochemical properties of doped MnO, MnO_2_ (*α*-, *δ*-, *β*-, *ε*-, *γ*-MnO_2_), Mn_2_O_3_, and Mn_3_O_4_ cathodes and the corresponding performance improvement mechanisms are summarized and analyzed. Specifically, doping engineering serves the following modification functions: (i) the phase transition and volume change in the cathodes can be alleviated, ensuring their structural stability throughout the charge/discharge cycles; (ii) defects can be introduced into the structure, thereby increasing the number of active sites for ion or proton storage; and (iii) the improvement in the transfer rate of ions and electrons and the weakening of the electrostatic repulsion between Zn^2+^ and MnO_2_ resulting from the lattice expansion and charge redistribution is beneficial for the insertion/de-insertion of Zn^2+^. Finally, this review outlines future research directions of Mn_x_O_y_ cathodes and AZIBs.

### 6.1. Study of Energy Storage Mechanisms

So far, representative storage mechanisms for Mn_x_O_y_ cathodes mainly include the Zn^2+^ intercalation/de-intercalation reaction, the Zn^2+^ and H^+^ co-intercalation/de-intercalation reaction, the chemical conversion reaction, dissolution/deposition, and hybrid reaction mechanisms. However, the energy storage mechanism of AZIBs is related to the composition, crystal structure, electrode morphology, electrolyte composition and concentration, and charging/discharging cycle times. An exact, reliable, and widely accepted mechanism of Mn_x_O_y_-based AZIBs still needs to be investigated. In situ characterization methods, including Raman diffraction, X-ray absorption diffraction, scanning and transmission electron microscopy, and electrochemical quartz crystal microbalance, enable the on-line monitoring of the phase and structural transformation of Mn_x_O_y_ cathodes during the charging/discharging process. DFT calculations offer insights into potential reactions at the atomic level. In addition, the high-throughput method involves the use of an automated operating system to perform the experimental procedure and the use of a sensitive and fast test instrument to collect experimental data. Therefore, the strategy of integrating high-throughput in situ characterization techniques with high-throughput DFT calculations can not only help to accurately and comprehensively understand the reaction mechanism of Mn_x_O_y_ cathodes but can also help to guide the design of suitable Mn_x_O_y_ cathodes to improve the performance of AZIBs.

### 6.2. Construction of Nanostructured Mn_x_O_y_

Nanostructures generally possess a significant specific surface area, high porosity, and high penetrability. As a result, they can mitigate the structural collapse induced by the volumetric swelling of the cathode in the process of electrochemical reaction, shorten the transport pathway of Zn^2+^ and electrons, and facilitate the insertion and extraction of Zn^2+^ ions. Nanostructures are classified into one-dimensional, two-dimensional, and three-dimensional structures. A one-dimensional structure with a micron size in the radial direction serves as an effective channel for current collection. In contrast, the ultrathin thickness and ultra-large exposed area of a two-dimensional structure can facilitate the charge transfer, shorten the ion transport path, and provide more reactive sites. The excellent volume density and rich pores of a three-dimensional structure can provide abundant ion adsorption sites, sufficient volume change buffering areas, and high electrolyte permeability, effectively avoiding self-aggregation and the side reactions of cathodes.

### 6.3. Optimization of Doping Engineering of Mn_x_O_y_

Currently, the doping engineering of Mn_x_O_y_ is mainly focused on either metal cation or non-metal anion doping. Anion doping refers to the replacement of oxygen with metal elements of low electronegativity, accompanied by the generation of lattice vacancies, while cation doping involves the replacement of manganese with metal elements that can expand the lattice spacing, alleviate the crystal stress, modify the electronic property, and promote the insertion of Zn^2+^. Therefore, it is reasonable to expect that the simultaneous incorporation of metal cations and non-metal anions into Mn_x_O_y_ could yield remarkable synergistic effects. In addition, there is still a lack of research on the precise control of doping concentration and sites. Next, it is of great importance to explore the intrinsic relationship between the type, concentration, and insertion site of doping elements and electrode kinetics in order to prepare high-performance doped Mn_x_O_y_ cathodes and enhance the electrochemical characteristic of AZIBs.

### 6.4. Practical Challenges and Limitations of Doping Mn_x_O_y_ for AZIBs

Despite that many achievements have been made in doping Mn_x_O_y_ for AZIBs in previous studies, there are still two challenges that need to be overcome to facilitate the large-scale and practical development of doping Mn_x_O_y_ for AZIBs. The first is to develop feasible and inexpensive methods to prepare doped Mn_x_O_y_ materials. Although many methods (e.g., hydrothermal method, co-precipitation) have successfully prepared doped Mn_x_O_y_ materials and displayed excellent electrochemical performance, most of these reported methods are complex and expensive for large-scale production. Thus, developing a feasible method with precise control of the doping site could greatly accelerate the commercialization of AZIBs. The second is to increase the areal capacity of cathode materials. Recently reported doped Mn_x_O_y_ cathodes only demonstrated an areal capacity of 0.1–0.2 mAh cm^−2^ due to the relatively low mass loading, which is far below the commercial standard (>2 mAh cm^−2^). Therefore, mass loadings higher than 10 mg cm^−2^ are urgently needed for the investigation of practical AZIBs.

### 6.5. Application of Doped Mn_x_O_y_-Based ZIBs in Flexible Storage Field

The instant development of flexible storage and the market-oriented utilization of portable electronic installations have promoted the development of flexible ZIBs with low cost and excellent bending rate tensile strength and environmental friendliness. On the one hand, the poor conductivity and lower specific capacity of Mn_x_O_y_ cathodes hinder their further development in flexible ZIBs. Therefore, it is urgent to modify Mn_x_O_y_ in order to boost the electrochemical characteristic. Additionally, aqueous batteries are unsuitable for flexible energy storage due to the evaporation and leakage of the aqueous electrolyte during the redox cycle. Therefore, the development of solid or gel electrolytes with good ductility, a high mechanical strength, and a wide operating temperature range and voltage window is of great significance for the commercialization of flexible ZIBs.

## Data Availability

No new data were created or analyzed in this study. Data sharing is not applicable to this article.

## References

[1] Hasapis D., Savvakis N., Tsoutsos T., Kalaitzakis K., Psychis S., Nikolaidis N.P. (2017). Design of large scale prosuming in Universities: The solar energy vision of the TUC campus. Energy Build..

[2] Li S., Tian Q., Chen J., Chen Y., Guo P., Wei C., Cui P., Jiang J., Li X., Xu Q. (2023). An intrinsically non-flammable organic electrolyte for wide temperature range supercapacitors. Chem. Eng. J..

[3] Li A., Wei Z., Wang Y., Zhang Y., Wang M., Zhang H., Ma Y., Liu C., Zou J., Ge B. (2023). Flexible quasi-3D zinc ion microcapacitor based on V_2_O_5_-PANI cathode and MXene anode. Chem. Eng. J..

[4] Zhang L., Wei C., Gao L., Lin M.-F., Eh A.L.-S., Chen J., Li S. (2024). Recent advances in electrospun nanostructured electrodes in zinc-ion batteries. Batteries.

[5] Barzin R., Chen J.J., Young B.R., Farid M.M. (2015). Peak load shifting with energy storage and price-based control system. Energy.

[6] Li X., Ma Y., Yue Y., Li G., Zhang C., Cao M., Xiong Y., Zou J., Zhou Y., Gao Y. (2022). A flexible Zn-ion hybrid micro-supercapacitor based on MXene anode and V_2_O_5_ cathode with high capacitance. Chem. Eng. J..

[7] Chen Y., Li S., Chen J., Gao L., Guo P., Wei C., Fu J., Xu Q. (2024). Sulfur-bridged bonds enabled structure modulation and space confinement of MnS for superior sodium-ion capacitors. J. Colloid Interface Sci..

[8] Gao L., Ma Y., Cao M. (2024). Self-supported Se-doped Na_2_Ti_3_O_7_ arrays for high performance sodium ion batteries. Int. J. Hydrogen Energy.

[9] Wang Q., Sarkar A., Wang D., Velasco L., Azmi R., Bhattacharya S.S., Bergfeldt T., Düvel A., Heitjans P., Brezesinski T. (2019). Multi-anionic and-cationic compounds: New high entropy materials for advanced Li-ion batteries. Energy Environ. Sci..

[10] Zhang M., Song X., Ou X., Tang Y. (2019). Rechargeable batteries based on anion intercalation graphite cathodes. Energy Storage Mater..

[11] Li H., Yang H., Sun Z., Shi Y., Cheng H.-M., Li F. (2019). A highly reversible Co_3_S_4_ microsphere cathode material for aluminum-ion batteries. Nano Energy.

[12] Olivetti E.A., Ceder G., Gaustad G.G., Fu X. (2017). Lithium-ion battery supply chain considerations: Analysis of potential bottlenecks in critical metals. Joule.

[13] Alias N., Mohamad A.A. (2015). Advances of aqueous rechargeable lithium-ion battery: A review. J. Power Sources.

[14] Kim H., Hong J., Park K.-Y., Kim H., Kim S.-W., Kang K. (2014). Aqueous rechargeable Li and Na ion batteries. Chem. Rev..

[15] Su D., McDonagh A., Qiao S.-Z., Wang G. (2017). High-capacity aqueous potassium-ion batteries for large-scale energy storage. Adv. Mater..

[16] Suo L., Borodin O., Sun W., Fan X., Yang C., Wang F., Gao T., Ma Z., Schroeder M., von Cresce A. (2016). Advanced high-voltage aqueous lithium-ion battery enabled by “water-in-bisalt” electrolyte. Angew. Chem..

[17] Liu H., Wang J.-G., You Z., Wei C., Kang F., Wei B. (2021). Rechargeable aqueous zinc-ion batteries: Mechanism, design strategies and future perspectives. Mater. Today.

[18] Kao-ian W., Mohamad A.A., Liu W.R., Pornprasertsuk R., Siwamogsatham S., Kheawhom S. (2022). Stability enhancement of zinc-ion batteries using non-aqueous electrolytes. Batter. Supercaps.

[19] Zhang L., Chen Y., Jiang Z., Chen J., Wei C., Wu W., Li S., Xu Q. (2024). Cation-anion redox active organic complex for high performance aqueous zinc ion battery. Energy Environ. Mater..

[20] Li L., Zhang W., Pan W., Wang M., Zhang H., Zhang D., Zhang D. (2021). Application of expanded graphite-based materials for rechargeable batteries beyond lithium-ions. Nanoscale.

[21] Bai Y., Zhang H., Tahir M.U., Xiang B. (2022). Conductive copper glue constructs a reversible and stable zinc metal anode interface for advanced aqueous zinc ion battery. J. Colloid Interface Sci..

[22] Xue T., Fan H.J. (2021). From aqueous Zn-ion battery to Zn-MnO_2_ flow battery: A brief story. J. Energy Chem..

[23] Bai M.-X., Gao J.-F., He Z.-H., Hou J.-F., Kong L.-B. (2022). Brookite phase vanadium dioxide (B) with nanosheet structure for superior rate capability aqueous Zn-ion batteries. J. Electroanal. Chem..

[24] Zampardi G., La Mantia F. (2020). Prussian blue analogues as aqueous Zn-ion batteries electrodes: Current challenges and future perspectives. Curr. Opin. Electrochem..

[25] Xu D., Zhang H., Zhou L., Gao X., Lu X. (2022). Structural regulation strategies towards high performance organic materials for next generation aqueous Zn-based batteries. ChemPhysMater.

[26] Lee W.S.V., Xiong T., Wang X., Xue J. (2021). Unraveling MoS_2_ and transition metal dichalcogenides as functional zinc-ion battery cathode: A perspective. Small Methods.

[27] Chao D., Zhou W., Ye C., Zhang Q., Chen Y., Gu L., Davey K., Qiao S.Z. (2019). An electrolytic Zn–MnO_2_ battery for high-voltage and scalable energy storage. Angew. Chem..

[28] Zhang D., Cao J., Zhang X., Insin N., Wang S., Han J., Zhao Y., Qin J., Huang Y. (2021). Inhibition of manganese dissolution in Mn_2_O_3_ cathode with controllable Ni^2+^ incorporation for high-performance zinc ion battery. Adv. Funct. Mater..

[29] Tan Q., Li X., Zhang B., Chen X., Tian Y., Wan H., Zhang L., Miao L., Wang C., Gan Y. (2020). Valence engineering via in situ carbon reduction on octahedron sites Mn_3_O_4_ for ultra-long cycle life aqueous Zn-ion battery. Adv. Energy Mater..

[30] Wang D., Lv H., Hussain T., Yang Q., Liang G., Zhao Y., Ma L., Li Q., Li H., Dong B. (2021). A manganese hexacyanoferrate framework with enlarged ion tunnels and two-species redox reaction for aqueous Al-ion batteries. Nano Energy.

[31] Tian B., Zhao W., Cui Y., Chu H., Qi S., Wang J., Xin B. (2022). Utilizing waste Zn-Mn batteries in combination with waste SCR catalyst to construct a magnetically recoverable and highly photocatalytic materials. Chem. Phys. Lett..

[32] Li X., Cheng H., Hu H., Pan K., Yuan T., Xia W. (2021). Recent advances of vanadium-based cathode materials for zinc-ion batteries. Chin. Chem. Lett..

[33] Jia X., Liu C., Neale Z.G., Yang J., Cao G. (2020). Active materials for aqueous zinc ion batteries: Synthesis, crystal structure, morphology, and electrochemistry. Chem. Rev..

[34] Lim M.B., Lambert T.N., Chalamala B.R. (2021). Rechargeable alkaline zinc–manganese oxide batteries for grid storage: Mechanisms, challenges and developments. Mater. Sci. Eng. R Rep..

[35] Zhao Y., Zhang P., Liang J., Xia X., Sun X. (2022). Uncovering Sulfur Doping Effect in MnO_2_ Nanosheets as an Efficient Cathode for Aqueous Zinc Ion Battery. Energy Storage Mater..

[36] Alfaruqi M.H., Gim J., Kim S., Song J., Jo J., Kim S., Mathew V., Kim J. (2015). Enhanced reversible divalent zinc storage in a structurally stable *α*-MnO_2_ nanorod electrode. J. Power Sources.

[37] Shi W., Lee W.S.V., Xue J. (2021). Recent development of Mn-based oxides as zinc-ion battery cathode. ChemSusChem.

[38] Jia S., Li L., Shi Y., Wang C., Cao M., Ji Y., Zhang D. (2024). Recent development of manganese dioxide-based materials as zinc-ion battery cathode. Nanoscale.

[39] Xu Y., Zhang G., Liu J., Zhang J., Wang X., Pu X., Wang J., Yan C., Cao Y., Yang H. (2023). Recent advances on challenges and strategies of manganese dioxide cathodes for aqueous zinc-ion batteries. Energy Environ. Mater..

[40] Luan J., Yuan H., Liu J., Zhong C. (2024). Recent advances on charge storage mechanisms and optimization strategies of Mn-based cathode in zinc–manganese oxides batteries. Energy Storage Mater..

[41] Li Q., Zhao Y., Wang Y., Khasraw A.K., Zhao Y., Sun X. (2023). Rational design of nanostructured MnO_2_ cathode for high-performance aqueous zinc ion batteries. Chem. Res. Chin. Univ..

[42] Li J., Ruan P., Chen X., Lei S., Lu B., Chen Z., Zhou J. (2023). Aqueous batteries for human body electronic devices: Focus Review. ACS Energy Lett..

[43] Zhang N., Wang J.-C., Guo Y.-F., Wang P.-F., Zhu Y.-R., Yi T.-F. (2023). Insights on rational design and energy storage mechanism of Mn-based cathode materials towards high performance aqueous zinc-ion batteries. Coord. Chem. Rev..

[44] Lin S., Zhang T. (2023). Review on recent developments, challenges, and perspectives of Mn-based oxide cathode materials for aqueous zinc-ion batteries and the status of Mn resources in China. Energy Fuels.

[45] Wang J., Szabo L., Madhav D., Ferreira I., Vandeginste V. (2023). Recent progress in interfacial engineering strategies for Mn-based oxide cathodes in aqueous zinc-ion batteries: Mechanisms, modifications, and performance enhancement. Energy Storage Mater..

[46] Zhao Y., Zhu Y., Zhang X. (2020). Challenges and perspectives for manganese-based oxides for advanced aqueous zinc-ion batteries. InfoMat.

[47] Xiong T., Zhang Y., Lee W.S.V., Xue J. (2020). Defect engineering in manganese-based oxides for aqueous rechargeable zinc-ion batteries: A review. Adv. Energy Mater..

[48] Gao X., Zhang H., Liu X., Lu X. (2020). Flexible Zn-ion batteries based on manganese oxides: Progress and prospect. Carbon Energy.

[49] Heng Y., Gu Z., Guo J., Wu X. (2021). Research progresses on vanadium-based cathode materials for aqueous zinc-ion batteries. Acta Phys. Chim. Sin..

[50] Lin M., Shao F., Tang Y., Lin H., Xu Y., Jiao Y., Chen J. (2022). Layered Co doped MnO_2_ with abundant oxygen defects to boost aqueous zinc-ion storage. J. Colloid Interface Sci..

[51] Yu B., Lu L., He Y., Dai X., Wang Y., Wang T., Chong S., Liu L., Liu Y., Tan Q. (2024). Hierarchical porous CS@ Ce-MnO_2_ as cathode for energy-dense and long-cycling flexible aqueous zinc-ion batteries. J. Colloid Interface Sci..

[52] Ko W.Y., Lubis A.L., Wang H.Y., Wu T.C., Lin S.T., Lin K.J. (2022). Facile construction of Zn-doped Mn_3_O_4_− MnO_2_ vertical nanosheets for aqueous zinc-ion battery cathodes. ChemElectroChem.

[53] He B., Huang J., Ji P., Hoang T.K., Han M., Li L., Zhang L., Gao Z., Ma J., Zhi J. (2023). Al doped manganous oxide for high-performance aqueous Zn-ion batteries. J. Power Sources.

[54] Kim H., Min K., Shim S.E., Lim D., Baeck S.-H. (2022). Ni-doped Mn_2_O_3_ microspheres as highly efficient electrocatalyst for oxygen reduction reaction and Zn-air battery. Int. J. Hydrogen Energy.

[55] Chen X., Ruan P., Wu X., Liang S., Zhou J. (2022). Crystal structures, reaction mechanisms, and optimization strategies of MnO_2_ cathode for aqueous rechargeable zinc batteries. Acta Phys. Chim. Sin..

[56] Huang J., Wang Z., Hou M., Dong X., Liu Y., Wang Y., Xia Y. (2018). Polyaniline-intercalated manganese dioxide nanolayers as a high-performance cathode material for an aqueous zinc-ion battery. Nat. Commun..

[57] Xu C., Li B., Du H., Kang F. (2012). Energetic zinc ion chemistry: The rechargeable zinc ion battery. Angew. Chem. Int. Ed..

[58] Alfaruqi M.H., Mathew V., Gim J., Kim S., Song J., Baboo J.P., Choi S.H., Kim J. (2015). Electrochemically induced structural transformation in a *γ*-MnO_2_ cathode of a high capacity zinc-ion battery system. Chem. Mater..

[59] Liang W., Che Y., Cai Z., Tang R., Ma Z., Zheng X., Wu X., Li J., Jin H., Zhu C. (2024). Surface decoration manipulating Zn^2+^/H^+^ carrier ratios for hyperstable aqueous zinc ion battery cathode. Adv. Funct. Mater..

[60] Li C., Yuan H., Liu T., Zhang R., Zhu J., Cui H., Wang Y., Cao D., Wang D., Zhi C. (2024). Distinguish MnO_2_/Mn^2+^ conversion/Zn^2+^ intercalation/H^+^ conversion chemistries at different potentials in aqueous Zn||MnO_2_ batteries. Angew. Chem..

[61] Sun W., Wang F., Hou S., Yang C., Fan X., Ma Z., Gao T., Han F., Hu R., Zhu M. (2017). Zn/MnO_2_ battery chemistry with H^+^ and Zn^2+^ coinsertion. J. Am. Chem. Soc..

[62] Jin Y., Zou L., Liu L., Engelhard M.H., Patel R.L., Nie Z., Han K.S., Shao Y., Wang C., Zhu J. (2019). Joint charge storage for high-rate aqueous zinc–manganese dioxide batteries. Adv. Mater..

[63] Li Y., Wang S., Salvador J.R., Wu J., Liu B., Yang W., Yang J., Zhang W., Liu J., Yang J. (2019). Reaction mechanisms for long-life rechargeable Zn/MnO_2_ batteries. Chem. Mater..

[64] Gao X., Wu H., Li W., Tian Y., Zhang Y., Wu H., Yang L., Zou G., Hou H., Ji X. (2020). H^+^-insertion boosted *α*-MnO_2_ for an aqueous Zn-ion battery. Small.

[65] Fang G., Zhu C., Chen M., Zhou J., Tang B., Cao X., Zheng X., Pan A., Liang S. (2019). Suppressing manganese dissolution in potassium manganate with rich oxygen defects engaged high-energy-density and durable aqueous zinc-ion battery. Adv. Funct. Mater..

[66] Xie S., Li X., Li Y., Liang Q., Dong L. (2022). Material design and energy storage mechanism of Mn-based cathodes for aqueous zinc-ion batteries. Chem. Rec..

[67] Hao J., Yuan L., Johannessen B., Zhu Y., Jiao Y., Ye C., Xie F., Qiao S.Z. (2021). Studying the conversion mechanism to broaden cathode options in aqueous zinc-ion batteries. Angew. Chem..

[68] Liu W., Zhang X., Huang Y., Jiang B., Chang Z., Xu C., Kang F. (2021). *β*-MnO_2_ with proton conversion mechanism in rechargeable zinc ion battery. J. Energy Chem..

[69] Kang J., Zhao Z., Li H., Meng Y., Hu B., Lu H. (2022). An overview of aqueous zinc-ion batteries based on conversion-type cathodes. Energy Mater..

[70] Lee B., Seo H.R., Lee H.R., Yoon C.S., Kim J.H., Chung K.Y., Cho B.W., Oh S.H. (2016). Critical role of pH evolution of electrolyte in the reaction mechanism for rechargeable zinc batteries. ChemSusChem.

[71] Yang J., Cao J., Peng Y., Yang W., Barg S., Liu Z., Kinloch I.A., Bissett M.A., Dryfe R.A. (2020). Unravelling the mechanism of rechargeable aqueous Zn–MnO_2_ batteries: Implementation of charging process by electrodeposition of MnO_2_. ChemSusChem.

[72] Liang R., Fu J., Deng Y.-P., Pei Y., Zhang M., Yu A., Chen Z. (2021). Parasitic electrodeposition in Zn-MnO_2_ batteries and its suppression for prolonged cyclability. Energy Storage Mater..

[73] Liu Z., Yang Y., Liang S., Lu B., Zhou J. (2021). pH-buffer contained electrolyte for self-adjusted cathode-free Zn–MnO_2_ batteries with coexistence of dual mechanisms. Small Struct..

[74] Moon H., Ha K.H., Park Y., Lee J., Kwon M.S., Lim J., Lee M.H., Kim D.H., Choi J.H., Choi J.H. (2021). Direct proof of the reversible dissolution/deposition of Mn^2+^/Mn^4+^ for mild-acid Zn-MnO_2_ batteries with porous carbon interlayers. Adv. Sci..

[75] Oberholzer P., Tervoort E., Bouzid A., Pasquarello A., Kundu D. (2018). Oxide versus nonoxide cathode materials for aqueous Zn batteries: An insight into the charge storage mechanism and consequences thereof. ACS Appl. Mater. Interfaces.

[76] Soundharrajan V., Sambandam B., Kim S., Islam S., Jo J., Kim S., Mathew V., Sun Y.-k., Kim J. (2020). The dominant role of Mn^2+^ additive on the electrochemical reaction in ZnMn_2_O_4_ cathode for aqueous zinc-ion batteries. Energy Storage Mater..

[77] Guo X., Zhou J., Bai C., Li X., Fang G., Liang S. (2020). Zn/MnO_2_ battery chemistry with dissolution-deposition mechanism. Mater. Today Energy.

[78] Ni Z., Liang X., Zhao L., Zhao H., Ge B., Li W. (2022). Tin doping manganese dioxide cathode materials with the improved stability for aqueous zinc-ion batteries. Mater. Chem. Phys..

[79] Chen C., Shi M., Zhao Y., Yang C., Zhao L., Yan C. (2021). Al-Intercalated MnO_2_ cathode with reversible phase transition for aqueous Zn-Ion batteries. Chem. Eng. J..

[80] Ren L., Yu G., Xu H., Wang W., Jiang Y., Ji M., Li S. (2021). Doping-induced static activation of MnO_2_ cathodes for aqueous Zn-ion batteries. ACS Sustain. Chem. Eng..

[81] Li Y., Li X., Duan H., Xie S., Dai R., Rong J., Kang F., Dong L. (2022). Aerogel-structured MnO_2_ cathode assembled by defect-rich ultrathin nanosheets for zinc-ion batteries. Chem. Eng. J..

[82] Yang S., Zhang L., Luo M., Cui Y., Wang J., Zhao D., Yang C., Wang X., Cao B. (2023). Synergistic combination of a Co-doped *σ*-MnO_2_ cathode with an electrolyte additive for a high-performance aqueous zinc-ion battery. ChemPhysMater.

[83] Zhang Y., Deng S., Luo M., Pan G., Zeng Y., Lu X., Ai C., Liu Q., Xiong Q., Wang X. (2019). Defect promoted capacity and durability of N-MnO_2–x_ branch arrays via low-temperature NH^3^ treatment for advanced aqueous zinc ion batteries. Small.

[84] Song Y., Li J., Qiao R., Dai X., Jing W., Song J., Chen Y., Guo S., Sun J., Tan Q. (2022). Binder-free flexible zinc-ion batteries: One-step potentiostatic electrodeposition strategy derived Ce doped-MnO_2_ cathode. Chem. Eng. J..

[85] Zhang M., Wu W., Luo J., Zhang H., Liu J., Liu X., Yang Y., Lu X. (2020). A high-energy-density aqueous zinc–manganese battery with a La–Ca co-doped *ε*-MnO_2_ cathode. J. Mater. Chem. A.

[86] Zhang Z., Li S., Zhao B., Zhang X., Wang X., Wen Z., Ji S., Sun J. (2021). Joint influence of nitrogen doping and oxygen vacancy on manganese dioxide as a high-capacity cathode for zinc-ion batteries. J. Phys. Chem. C.

[87] Wu X., Zhou S., Li Y., Yang S., Xiang Y., Jiang J., Liu Z., Fan D., Zhang H., Zhu L. (2021). Na-containing manganese-based cathode materials synthesized by sol-gel method for zinc-based rechargeable aqueous battery. J. Alloys Compd. Interdiscip. J. Mater. Sci. Solid-State Chem. Phys..

[88] Jiao Y., Kang L., Berry-Gair J., McColl K., Li J., Dong H., Jiang H., Wang R., Corà F., Brett D.J. (2020). Enabling stable MnO_2_ matrix for aqueous zinc-ion battery cathodes. J. Mater. Chem. A.

[89] Xiong T., Yu Z.G., Wu H., Du Y., Xie Q., Chen J., Zhang Y.W., Pennycook S.J., Lee W.S.V., Xue J. (2019). Defect engineering of oxygen-deficient manganese oxide to achieve high-performing aqueous zinc ion battery. Adv. Energy Mater..

[90] Du M., Miao Z., Li H., Sang Y., Liu H., Wang S. (2021). Strategies of structural and defect engineering for high-performance rechargeable aqueous zinc-ion batteries. J. Mater. Chem. A.

[91] Jia H., Li Y., Fu L., Ali U., Liu B., Zhang L., Wang H., Li L., Wang H.G., Wang C. (2023). Ion pre-embedding engineering of *δ*-MnO_2_ for chemically self-charging aqueous zinc ions batteries. Small.

[92] Yang Z., Li B., Sun B., Jia D., Gao Z., Gao S. (2024). A mini review: Applications of pre-embedding active ion strategies in electrochemical energy storage systems. J. Electroanal. Chem..

[93] Zhao X., Mao L., Cheng Q., Liao F., Yang G., Lu X., Chen L. (2021). Interlayer engineering of preintercalated layered oxides as cathode for emerging multivalent metal-ion batteries: Zinc and beyond. Energy Storage Mater..

[94] Liu Y., Xu J., Li J., Yang Z., Huang C., Yu H., Zhang L., Shu J. (2022). Pre-intercalation chemistry of electrode materials in aqueous energy storage systems. Coord. Chem. Rev..

[95] Li G., Sun L., Zhang S., Zhang C., Jin H., Davey K., Liang G., Liu S., Mao J., Guo Z. (2024). Developing cathode materials for aqueous zinc ion batteries: Challenges and practical prospects. Adv. Funct. Mater..

[96] Shi X., Liu X., Wang E., Cao X., Yu Y., Cheng X., Lu X. (2023). Boosting the Zn ion storage ability of amorphous MnO_2_ via surface engineering and valence modulation. Carbon Neutralization.

[97] Panda M.R., El Meragawi S., Mirshekarloo M.S., Chen W., Shaibani M., Majumder M. (2024). Acidity-aided surface modification strategy to enhance in situ MnO_2_ deposition for high performance Zn-MnO_2_ battery prototypes. Small.

[98] Liao X., Pan C., Yan H., Zhu Y., Pan Y., Yin C. (2022). Polyaniline-functionalized graphene composite cathode with enhanced Zn^2+^ storage performance for aqueous zinc-ion battery. Chem. Eng. J..

[99] Yang Q., Li X., Chen Z., Huang Z., Zhi C. (2021). Cathode engineering for high energy density aqueous Zn batteries. Acc. Mater. Res..

[100] Galos J., Pattarakunnan K., Best A.S., Kyratzis I.L., Wang C.H., Mouritz A.P. (2021). Energy storage structural composites with integrated lithium-ion batteries: A review. Adv. Mater. Technol..

[101] Kamenskii M.A., Volkov F.S., Eliseeva S.N., Tolstopyatova E.G., Kondratiev V.V. (2023). Enhancement of electrochemical performance of aqueous zinc ion batteries by structural and interfacial design of MnO_2_ cathodes: The metal ion doping and introduction of conducting polymers. Energies.

[102] Qian H., Ren H., Zhang Y., He X., Li W., Wang J., Hu J., Yang H., Sari H.M.K., Chen Y. (2022). Surface doping vs. bulk doping of cathode materials for lithium-ion batteries: A review. Electrochem. Energy Rev..

[103] Yuan Y., Chen Z., Yu H., Zhang X., Shu J. (2020). Heteroatom-doped carbon-based materials for lithium and sodium ion batteries. Energy Storage Mater..

[104] Li Y., Chen M., Liu B., Zhang Y., Liang X., Xia X. (2020). Heteroatom doping: An effective way to boost sodium ion storage. Adv. Energy Mater..

[105] Zhang B., Chen J., Sun W., Shao Y., Zhang L., Zhao K. (2022). Challenges and perspectives for doping strategy for manganese-based zinc-ion battery cathode. Energies.

[106] Zhao Q., Song A., Ding S., Qin R., Cui Y., Li S., Pan F. (2020). Preintercalation strategy in manganese oxides for electrochemical energy storage: Review and prospects. Adv. Mater..

[107] Nam G., Hwang C., Jang H., Kane N., Ahn Y., Kwak M.J., Luo Z., Li T., Kim M.G., Liu N. (2024). Tuning proton insertion chemistry for sustainable aqueous zinc-ion batteries. Small.

[108] Young M.J., Holder A.M., George S.M., Musgrave C.B. (2015). Charge storage in cation incorporated *α*-MnO_2_. Chem. Mater..

[109] Hu Z., Xiao X., Huang L., Chen C., Li T., Su T., Cheng X., Miao L., Zhang Y., Zhou J. (2015). 2D vanadium doped manganese dioxides nanosheets for pseudocapacitive energy storage. Nanoscale.

[110] Radhamani A., Surendra M.K., Rao M.R. (2018). Zn doped *δ*-MnO_2_ nano flakes: An efficient electrode material for aqueous and solid state asymmetric supercapacitors. Appl. Surf. Sci..

[111] Nasser R., Zhang G.-F., Song J.-M. (2020). Facile and low-cost synthesis of cobalt-doped MnO_2_ decorated with graphene oxide for high performance 2.3 V aqueous asymmetric supercapacitors. Electrochim. Acta.

[112] Zhang H., Ye K., Huang X., Wang X., Cheng K., Xiao X., Wang G., Cao D. (2017). Preparation of Mg_1.1_Mn_6_O_12_· 4.5H_2_O with nanobelt structure and its application in aqueous magnesium-ion battery. J. Power Sources.

[113] Wu J., Raza W., Wang P., Hussain A., Ding Y., Yu J., Wu Y., Zhao J. (2022). Zn-doped MnO_2_ ultrathin nanosheets with rich defects for high performance aqueous supercapacitors. Electrochim. Acta.

[114] Bai H., Liang S., Wei T., Zhou Q., Shi M., Jiang Z., Feng J., Zhang M., Fan Z. (2022). Enhanced pseudo-capacitance and rate performance of amorphous MnO_2_ for supercapacitor by high Na doping and structural water content. J. Power Sources.

[115] Li W., Qin L., Liu Z., Li L., Li W., Fang G. (2024). Potassium-ion-doped manganese oxides and kaolinite electrolyte additives for aqueous zinc-ion batteries. ACS Appl. Nano Mater..

[116] Li H., Huang Z., Chen B., Jiang Y., Li C., Xiao W., Yan X. (2022). A high-performance MnO_2_ cathode doped with group Ⅷ metal for aqueous Zn-ion batteries: In-situ X-Ray diffraction study on Zn^2+^ storage mechanism. J. Power Sources.

[117] Hou X., Li C., Li M., Liu Y., Zhu W., Li Z., Xu Y. (2023). Synthesis of Cu-doped layered transition metal oxide cathode materials directly from metal-organic Frameworks for Sodium-Ion Batteries. Chin. J. Chem..

[118] Zheng Y., Li J., Ji S., Hui K.S., Wang S., Xu H., Wang K., Dinh D.A., Zha C., Shao Z. (2023). Zinc-doping strategy on P2-type Mn-based layered oxide cathode for high-performance potassium-ion batteries. Small.

[119] Liao Y., Chen H.-C., Yang C., Liu R., Peng Z., Cao H., Wang K. (2022). Unveiling performance evolution mechanisms of MnO_2_ polymorphs for durable aqueous zinc-ion batteries. Energy Storage Mater..

[120] Wu B., Zhang G., Yan M., Xiong T., He P., He L., Xu X., Mai L. (2018). Graphene scroll-coated *α*-MnO_2_ nanowires as high-performance cathode materials for aqueous Zn-ion battery. Small.

[121] Juran T.R., Young J., Smeu M. (2018). Density functional theory modeling of MnO_2_ polymorphs as cathodes for multivalent ion batteries. J. Phys. Chem. C.

[122] Islam S., Alfaruqi M.H., Mathew V., Song J., Kim S., Kim S., Jo J., Baboo J.P., Pham D.T., Putro D.Y. (2017). Facile synthesis and the exploration of the zinc storage mechanism of *β*-MnO_2_ nanorods with exposed (101) planes as a novel cathode material for high performance eco-friendly zinc-ion batteries. J. Mater. Chem. A.

[123] Kwon N.H., Lee K.-G., Kim H.K., Hwang S.-J. (2021). MnO_2_-based nanostructured materials for various energy applications. Mater. Chem. Front..

[124] Worku A.K., Ayele D.W., Habtu N.G., Teshager M.A., Workineh Z.G. (2021). Recent progress in MnO_2_-based oxygen electrocatalysts for rechargeable zinc-air batteries. Mater. Today Sustain..

[125] Guo X., Yang S., Wang D., Chen A., Wang Y., Li P., Liang G., Zhi C. (2021). The energy storage mechanisms of MnO_2_ in batteries. Curr. Opin. Electrochem..

[126] Guo D., Zhao W., Pan F., Liu G. (2022). Block copolymer-derived porous carbon fibers enable high MnO_2_ loading and fast charging in aqueous zinc-ion battery. Batter. Supercaps.

[127] Wei C., Xu C., Li B., Du H., Kang F. (2012). Preparation and characterization of manganese dioxides with nano-sized tunnel structures for zinc ion storage. J. Phys. Chem. Solids.

[128] Xu J.-W., Gao Q.-L., Xia Y.-M., Lin X.-S., Liu W.-L., Ren M.-M., Kong F.-G., Wang S.-J., Lin C. (2021). High-performance reversible aqueous zinc-ion battery based on iron-doped alpha-manganese dioxide coated by polypyrrole. J. Colloid Interface Sci..

[129] Li Q., Wang C., Zhu Y., Du W., Liu W., Yao M., Wang Y., Qian Y., Feng S. (2024). Unlocking the critical role of Mg doping in *α*-MnO_2_ cathode for aqueous zinc ion batteries. Chem. Eng. J..

[130] Zhang L., Liao Y., Ye M., Cai W., Xiao M., Hu C., Zhong B., Wan F., Guo X. (2023). Regeneration of spent lithium manganate batteries into Al-doped MnO_2_ cathodes toward aqueous Zn batteries. ACS Appl. Mater. Interfaces.

[131] Gao L., Hu H., Zhang C., Cao M. (2024). Gallium regulated MnO_2_ toward high performance Zn ion batteries. Vacuum.

[132] Ma Y., Xu M., Liu R., Xiao H., Liu Y., Wang X., Huang Y., Yuan G. (2022). Molecular tailoring of MnO_2_ by bismuth doping to achieve aqueous zinc-ion battery with capacitor-level durability. Energy Storage Mater..

[133] Le T., Takeuchi E.S., Takeuchi K.J., Marschilok A.C., Liu P. (2023). Tuning discharge behavior of hollandite *α*-MnO_2_ in hydrated zinc ion battery by transition metal substitution. J. Phys. Chem. C.

[134] Li Y., Liu X., Ji T., Zhang M., Yan X., Yao M., Sheng D., Li S., Ren P., Shen Z. (2024). Potassium ion doped manganese oxide nanoscrolls enhanced the performance of aqueous zinc-ion batteries. Chin. Chem. Lett..

[135] Lan R., Gkanas E., Sahib A.J.S., Greszta A., Bhagat R., Roberts A. (2024). The effect of copper doping in *α*-MnO_2_ as cathode material for aqueous Zinc-ion batteries. J. Alloys Compd..

[136] Wang C., Yang H., Wang B., Ding P., Wan Y., Bao W., Li Y., Ma S., Liu Y., Lu Y. (2023). Dual cation doping enabling simultaneously boosted capacity and rate capability of MnO_2_ cathodes for Zn//MnO_2_ batteries. Nano Res..

[137] Alfaruqi M.H., Islam S., Mathew V., Song J., Kim S., Tung D.P., Jo J., Kim S., Baboo J.P., Xiu Z. (2017). Ambient redox synthesis of vanadium-doped manganese dioxide nanoparticles and their enhanced zinc storage properties. Appl. Surf. Sci..

[138] Zhang R., Liang P., Yang H., Min H., Niu M., Jin S., Jiang Y., Pan Z., Yan J., Shen X. (2022). Manipulating intercalation-extraction mechanisms in structurally modulated *δ*-MnO_2_ nanowires for high-performance aqueous zinc-ion batteries. Chem. Eng. J..

[139] Wang H., Liang M., Gao J., Ma C., He Z., Zhao Y., Miao Z. (2022). Robust structural stability of flower-like *δ*-MnO_2_ as cathode for aqueous zinc ion battery. Colloids Surf. A Physicochem. Eng. Asp..

[140] Xie Q., Cheng G., Xue T., Huang L., Chen S., Sun Y., Sun M., Wang H., Yu L. (2022). Alkali ions pre-intercalation of *δ*-MnO_2_ nanosheets for high-capacity and stable Zn-ion battery. Mater. Today Energy.

[141] Yan L., Liu B., Hao J., Han Y., Zhu C., Liu F., Zou X., Zhou Y., Xiang B. (2023). In–situ cation–inserted MnO_2_ with selective accelerated intercalation of individual H^+^ or Zn^2+^ ions in aqueous zinc ion batteries. J. Energy Chem..

[142] Xia J., Zhou Y., Zhang J., Lu T., Gong W., Zhang D., Wang X., Di J. (2023). Triggering high capacity and superior reversibility of manganese oxides cathode via magnesium modulation for Zn//MnO_2_ batteries. Small.

[143] Long F., Xiang Y., Yang S., Li Y., Du H., Liu Y., Wu X., Wu X. (2022). Layered manganese dioxide nanoflowers with Cu^2+^ and Bi^3+^ intercalation as high-performance cathode for aqueous zinc-ion battery. J. Colloid Interface Sci..

[144] Zhao W., Fee J., Khanna H., March S., Nisly N., Rubio S.J.B., Cui C., Li Z., Suib S.L. (2022). A two-electron transfer mechanism of the Zn-doped *δ*-MnO_2_ cathode toward aqueous Zn-ion batteries with ultrahigh capacity. J. Mater. Chem. A.

[145] Xia X., Zhao Y., Zhao Y., Xu M., Liu W., Sun X. (2023). Mo doping provokes two electron reaction in MnO_2_ with ultrahigh capacity for aqueous zinc ion batteries. Nano Res..

[146] Pu X., Li X., Wang L., Maleki Kheimeh Sari H., Li J., Xi Y., Shan H., Wang J., Li W., Liu X. (2022). Enriching oxygen vacancy defects via Ag–O–Mn bonds for enhanced diffusion kinetics of *δ*-MnO_2_ in zinc-ion batteries. ACS Appl. Mater. Interfaces.

[147] Wang D., Liu Z., Gao X.-W., Gu Q., Zhao L., Luo W.-B. (2023). Massive anionic fluorine substitution two-dimensional *δ*-MnO_2_ nanosheets for high-performance aqueous zinc-ion battery. J. Energy Storage.

[148] Sun Y., Zhuang S., Ren Y., Jiang S., Pan X., Sun G., Zhu B., Wen Y., Li X., Tu F. (2023). Promoting cycle stability and rate performance of birnessite-type MnO_2_ cathode via cupper and bismuth dual ions pre-intercalation for aqueous zinc-ion batteries. J. Energy Storage.

[149] Ding X., Wen Y., Qing C., Wei Y., Wang P., Liu J., Peng Z., Song Y., Chen H., Rong Q. (2024). Cr-induced enhancement of structural stability in *δ*-MnO_2_ for aqueous Zn-ion batteries. J. Alloys Compd..

[150] Zhao Y., Xia X., Li Q., Wang Y., Fan Y., Zhao Y., Liu W., Sun X. (2024). Activating the redox chemistry of MnO_2_/Mn^2+^ in aqueous Zn batteries based on multi-ions doping regulation. Energy Storage Mater..

[151] Han M., Huang J., Liang S., Shan L., Xie X., Yi Z., Wang Y., Guo S., Zhou J. (2020). Oxygen defects in *β*-MnO_2_ enabling high-performance rechargeable aqueous zinc/manganese dioxide battery. iScience.

[152] Deng Z., Huang J., Liu J., Ren L., Zhu L., Xiao X., Tan M. (2019). *β*-MnO_2_ nanolayer coated on carbon cloth as a high-activity aqueous zinc-ion battery cathode with high-capacity and long-cycle-life. Mater. Lett..

[153] Han R., Pan Y., Du C., Xiang Y., Wang Y., Zhu H., Yin C. (2024). Eu doping *β*-MnO_2_ as cathode materials for high specific capacity aqueous zinc ion batteries. J. Energy Storage.

[154] Zhang Y., Liu Y., Liu Z., Wu X., Wen Y., Chen H., Ni X., Liu G., Huang J., Peng S. (2022). MnO_2_ cathode materials with the improved stability via nitrogen doping for aqueous zinc-ion batteries. J. Energy Chem..

[155] Zhang L., Tan B.C., Li W.P. (2021). Synthesis and electrochemical properties of Cu^2+^-doped MnO_2_ as cathode materials for aqueous zinc ion batteries. CIESC J..

[156] Ji J., Yao J., Xu Y., Wan H., Zhang B., Lv L., Li J., Wang N., Zheng Z., Zhang J. (2023). Promoting proton migration kinetics by Ni^2+^ regulating enables improved aqueous Zn-MnO_2_ batteries. Energy Environ. Mater..

[157] Chen Y., Hu X., Chen X., Liu J.-H., Huang Y., Cao D. (2023). Trimetallic-organic framework-derived Ni-doped MnO/PC as cathodes for high-performance aqueous zinc-ion batteries. Chem. Eng. J..

[158] Yu P., Zhou J., Zheng M., Li M., Hu H., Xiao Y., Liu Y., Liang Y. (2021). Boosting zinc ion energy storage capability of inert MnO cathode by defect engineering. J. Colloid Interface Sci..

[159] Zou R., Tang Z., Chen X., Li Z., Lei G. (2022). Exploration of calcium-doped manganese monoxide cathode for high-performance aqueous zinc-ion batteries. Energy Fuels.

[160] Sun K., Shen Y., Min J., Pang J., Zheng Y., Gu T., Wang G., Chen L. (2023). MOF-derived Zn/Co co-doped MnO/C microspheres as cathode and Ti_3_C_2_@ Zn as anode for aqueous zinc-ion full battery. Chem. Eng. J..

[161] Jia H., Li Y., Ali U., Liu B., Jin Z., Li L., Chen Y., Zhang L., Wang T., Wang C. (2024). High-entropy doping strategy towards reinforced Mn-O bond for durable aqueous zinc ion batteries. Nano Energy.

[162] Liu N., Wu X., Yin Y., Chen A., Zhao C., Guo Z., Fan L., Zhang N. (2020). Constructing the efficient ion diffusion pathway by introducing oxygen defects in Mn_2_O_3_ for high-performance aqueous zinc-ion batteries. ACS Appl. Mater. Interfaces.

[163] Komolov A., Lazneva E., Gerasimova N., Panina Y.A., Sobolev V., Koroleva A., Pshenichnyuk S., Asfandiarov N., Modelli A., Handke B. (2019). Conduction band electronic states of ultrathin layers of thiophene/phenylene co-oligomers on an oxidized silicon surface. J. Electron Spectrosc. Relat. Phenom..

[164] Pronin I.A., Averin I.A., Karmanov A.A., Yakushova N.D., Komolov A.S., Lazneva E.F., Sychev M.M., Moshnikov V.A., Korotcenkov G. (2022). Control over the surface properties of zinc oxide powders via combining mechanical, electron beam, and thermal processing. Nanomaterials.

[165] Saadi-motaallegh S., Javanbakht M., Omidvar H., Habibzadeh S. (2023). A Novel Ni-doped ZnMn_2_O_4_/Mn_2_O_3_ nanocomposite synthesized by pulsed potential as superior zinc ion battery cathode material. J. Alloys Compd..

[166] Karuppaiah M., Sakthivel P., Asaithambi S., Bharat L.K., Nagaraju G., Ahamad T., Balamurugan K., Yuvakkumar R., Ravi G. (2020). Elevated energy density and cycle stability of *α*-Mn_2_O_3_ 3D-microspheres with addition of neodymium dopant for pouch-type hybrid supercapacitors. Electrochim. Acta.

[167] Darjazi H., Davarani S.S.H., Moazami H.R., Yousefi T., Tabatabaei F. (2016). Evaluation of charge storage ability of chrome doped Mn_2_O_3_ nanostructures derived by cathodic electrodeposition. Prog. Nat. Sci. Mater. Int..

[168] Zhao F.F., Li C.Y., Li S.H., Wang B., Huang B.K., Hu K., Liu L.H., Yu W.W., Li H.Z. (2024). Continuous solar energy conversion windows integrating zinc anode-based electrochromic device and IoT system. Adv. Mater..

[169] Wang D., Zhang S., Li C., Chen X., Zhang W., Ge X., Lin H., Shi Z., Feng S. (2022). High-performance aqueous zinc-ion battery based on an Al_0.35_Mn_2.52_O_4_ cathode: A design strategy from defect engineering and atomic composition tuning. Small.

[170] Ji J., Wan H., Zhang B., Wang C., Gan Y., Tan Q., Wang N., Yao J., Zheng Z., Liang P. (2021). Co^2+^/^3+^/^4+^-Regulated electron state of Mn-O for superb aqueous zinc-manganese oxide batteries. Adv. Energy Mater..

[171] Li D., Wang Z.-R., Xia Y.-M., Gao Q.-L., Ren M.-M., Liu W.-L., Kong F.-G., Wang S.-J., Li S.-H. (2021). Copper-doped manganese tetroxide composites with excellent electrochemical performance for aqueous zinc-ion batteries. J. Electroanal. Chem..

[172] Li S., Yao Y., Zhang Y., Gong Y., Wu M., Xue Y., Yang J., Li L. (2023). High-loading cobalt-doped manganese tetroxide on carbon cloth as an electrode material for high-performance zinc ion hybrid capacitors. Electrochim. Acta.

[173] Hong J.S., Seo H., Lee Y.H., Cho K.H., Ko C., Park S., Nam K.T. (2020). Nickel-doping effect on Mn_3_O_4_ nanoparticles for electrochemical water oxidation under neutral condition. Small Methods.

[174] Li T., Hu Y., Liu K., Yin J., Li Y., Fu G., Zhang Y., Tang Y. (2022). Hollow yolk-shell nanoboxes assembled by Fe-doped Mn_3_O_4_ nanosheets for high-efficiency electrocatalytic oxygen reduction in Zn-Air battery. Chem. Eng. J..

[175] Adoor P., Hegde S.S., Bhat B.R., Yethadka S.N., Yeenduguli R. (2023). Elucidating the role of copper-induced mixed phases on the electrochemical performance of Mn-based thin-film electrodes. ACS Omega.

